# Localization of the tubby domain, a PI(4,5)P_2_ biosensor, to E-Syt3-rich endoplasmic reticulum–plasma membrane junctions

**DOI:** 10.1242/jcs.260848

**Published:** 2023-08-04

**Authors:** Veronika Thallmair, Lea Schultz, Saskia Evers, Theresa Jolie, Christian Goecke, Michael G. Leitner, Sebastian Thallmair, Dominik Oliver

**Affiliations:** ^1^ Department of Neurophysiology, Institute of Physiology and Pathophysiology, Philipps University Marburg, 35037 Marburg, Germany; ^2^ DFG Research Training Group, Membrane Plasticity in Tissue Development and Remodeling, GRK 2213, Philipps University Marburg, 35037 Marburg, Germany; ^3^ Institute of Physiology, Department of Physiology and Medical Physics, Medical University of Innsbruck, 6020 Innsbruck, Austria; ^4^ Drug Discovery Sciences, Boehringer Ingelheim Pharma GmbH&Co.KG, Birkendorfer Str. 65, 88400 Biberach an der Riß, Germany; ^5^ Frankfurt Institute for Advanced Studies, 60438 Frankfurt am Main, Germany; ^6^ Groningen Biomolecular Sciences and Biotechnology Institute and The Zernike Institute for Advanced Material, University of Groningen, 9747 AG Groningen, The Netherlands; ^7^ Center for Mind, Brain and Behavior (CMBB), Universities of Marburg and Giessen, 35032 Marburg, Germany

**Keywords:** ER–PM junction, Fluorescence imaging, Lipid-binding domain, Molecular dynamics simulations, Phosphoinositide signaling

## Abstract

The phospholipid phosphatidylinositol (4,5)-bisphosphate [PI(4,5)P2] acts as a signaling lipid at the plasma membrane (PM) with pleiotropic regulatory actions on multiple cellular processes. Signaling specificity might result from spatiotemporal compartmentalization of the lipid and from combinatorial binding of PI(4,5)P_2_ effector proteins to additional membrane components. Here, we analyzed the spatial distribution of tubbyCT, a paradigmatic PI(4,5)P_2_-binding domain, in live mammalian cells by total internal reflection fluorescence (TIRF) microscopy and molecular dynamics simulations. We found that unlike other well-characterized PI(4,5)P_2_ recognition domains, tubbyCT segregates into distinct domains within the PM. TubbyCT enrichment occurred at contact sites between PM and endoplasmic reticulum (ER) (i.e. at ER–PM junctions) as shown by colocalization with ER–PM markers. Localization to these sites was mediated in a combinatorial manner by binding to PI(4,5)P_2_ and by interaction with a cytosolic domain of extended synaptotagmin 3 (E-Syt3), but not other E-Syt isoforms. Selective localization to these structures suggests that tubbyCT is a novel selective reporter for a ER–PM junctional pool of PI(4,5)P_2_. Finally, we found that association with ER–PM junctions is a conserved feature of tubby-like proteins (TULPs), suggesting an as-yet-unknown function of TULPs.

## Teaser

**Summary:** The PI(4,5)P_2_-binding domain tubbyCT is recruited into ER–PM junctions through coincidence detection of PI(4,5)P_2_ and extended synaptotagmin 3 (E-Syt3).

## INTRODUCTION

Phosphatidylinositol (4,5)-bisphosphate [PI(4,5)P_2_] is a relatively scarce but essential component of the inner leaflet of the plasma membrane (PM). Despite its low abundance, it is an important determinant of PM identity. PI(4,5)P_2_ acts via recruitment and regulation of cytosolic or membranous proteins, and thereby regulates copious cellular processes such as exocytosis and endocytosis, cytoskeletal anchorage and cell motility. It impacts strongly on ion flux through channels and transporters and functions as the substrate for signal-triggered generation of the second messengers phosphatidylinositol (3,4,5)-trisphosphate [PI(3,4,5)P_3_], inositol (1,4,5)-trisphosphate (IP_3_) and diacylglycerol (DAG) (reviewed in Di Paolo and De Camilli, 2006; Schink et al., 2016; Wills and Hammond, 2022). In order to rationalize specificity in controlling the plethora of different functions, PI(4,5)P_2_ has been postulated to exist in distinct pools partitioned through spatial compartmentalization or by metabolic channeling (reviewed in Grabon et al., 2015; Kwiatkowska, 2010).

Lateral inhomogeneity has been shown for many PM components – both lipids and proteins – and contributes strongly to membrane functionality. A prominent example for lipid-driven membrane compartmentalization is the concept of lipid rafts, densely packed liquid-ordered domains, separated from the liquid-disordered phase. The specific lipid composition then recruits particular sets of proteins, which in turn facilitate specific signaling pathways (Lingwood and Simons, 2010). Vice versa, proteins contribute to membrane lipid inhomogeneity as they maintain their specific lipid footprints, that is, they can dramatically change the lipid composition in their surroundings through hydrophobic and electrostatic interactions (Corradi et al., 2018; Epand, 2008; McLaughlin and Murray, 2005). Finally, diffusion barriers formed by proteins, together with local lipid synthesis or degradation, can shape distinct PM compartments (Caudron and Barral, 2009; Jacobson et al., 2019; Trimble and Grinstein, 2015). PI(4,5)P_2_ accumulation in membrane subdomains has been observed with a variety of different approaches (Fujita et al., 2009; Hope and Pike, 1996; van den Bogaart et al., 2011; Wang and Richards, 2012), and distinct PI(4,5)P_2_ pools have been attributed to different functionalities (Johnson et al., 2008). Also, PI(4,5)P_2_ metabolism has been shown to differ between the distinct pools (Fujita et al., 2009; Myeong et al., 2021). However, the topic has remained controversial. For example, the unrestricted diffusion of PI(4,5)P_2_ observed in the PM argues against spatial compartmentalization (Pacheco et al., 2023). Also many of the protocols applied to analyze lipid concentration inhomogeneities involve fixation and staining steps that could disturb the native distribution of membrane components (Hammond, 2016; van Rheenen et al., 2005).

In living cells, membrane lipids have been studied extensively through the use of fluorescently labeled lipid recognition protein domains that associate to membrane compartments containing their specific lipid ligand. Ideally, the localization of these biosensors should be driven only by their affinity to the respective lipid, such that spatial distribution and dynamics of the lipid are faithfully reflected by subcellular localization of the sensor. However, many lipid recognition domains are coincidence detectors; their membrane association is determined not only by a specific lipid ligand, but also by binding to a second interaction partner (Hammond and Balla, 2015). While limiting application as an unbiased and general lipid biosensor, coincidence detection can enable the study of a particular lipid pool. FAPP1 for example, a PI4P–Arf1 coincidence sensor has been used to image the Golgi-localized pool of PI4P, but fails to detect PM-localized PI4P (Godi et al., 2004; Hammond and Balla, 2015).

Few PI(4,5)P_2_ recognition domains have been characterized in detail and used as biosensors. The PH domain of phospholipase Cδ1 (PLCδ1-PH) has been used most broadly. Although it binds with high affinity to PI(4,5)P_2_, its affinity to IP_3_ is comparable, compromising its use in the context of PLC activity (Hirose et al., 1999; Nash et al., 2001; Nelson et al., 2008; van der Wal et al., 2001). Alternatively, the structurally unrelated C-terminal domain of the tubby protein (tubbyCT) has been used fairly frequently for imaging PI(4,5)P_2_ (e.g. Hackelberg and Oliver, 2018; Nelson et al., 2008; Quinn et al., 2008). In addition to a canonical PI(4,5)P_2_-binding pocket (Santagata et al., 2001), tubbyCT additionally holds an adjacent second binding site formed by a strongly basic patch (Thallmair et al., 2022). Consequently, tubbyCT binds to two PI(4,5)P_2_ molecules in a cooperative manner resulting in a steepened concentration dependency (Thallmair et al., 2022). Notably, lipid binding is specific to PI(4,5)P_2_ over other phosphoinositides, both in molecular dynamics simulations and experimentally in living cells (Halaszovich et al., 2009; Leitner et al., 2019; Thallmair et al., 2022). Despite these apparently straightforward binding properties, reported PM association–dissociation responses in living cells are remarkably variable and inconsistent with respect to presumed cellular PI(4,5)P_2_ concentration dynamics (Hackelberg and Oliver, 2018; Quinn et al., 2008; Santagata et al., 2001; Szentpetery et al., 2009). An explanation might be pool-selective binding to PI(4,5)P_2_, although neither the identity of a putative tubby-associated pool nor the mechanistic basis for such selectivity are known.

Given the demand for a full characterization of the available PI(4,5)P_2_ sensors, we examined the spatial distribution of tubbyCT by total internal reflection fluorescence (TIRF) microscopy. We observed strongly heterogeneous localization of tubbyCT in the PM of various cell types. Membrane patches with pronounced local enrichment colocalized with the tethering protein, extended synaptotagmin 3 (E-Syt3), indicating that tubbyCT accumulates in membrane contact sites (MCSs) between the endoplasmic reticulum (ER) and the PM (ER-PM junctions). Binding of tubbyCT to both PI(4,5)P_2_ and E-Syt3 promoted clustering, indicating that tubbyCT is a lipid–protein coincidence sensor biased towards the ER–PM junctional membrane. Using tubbyCT as an ER–PM junction-specific PI(4,5)P_2_ biosensor might provide new insights into local PI(4,5)P_2_ content and dynamics of these pivotal hubs of phosphoinositide homeostasis.

## RESULTS

### TubbyCT, but not other PI(4,5)P_2_-binding domains, preferentially localizes to PM subdomains

**Fig. 1.**
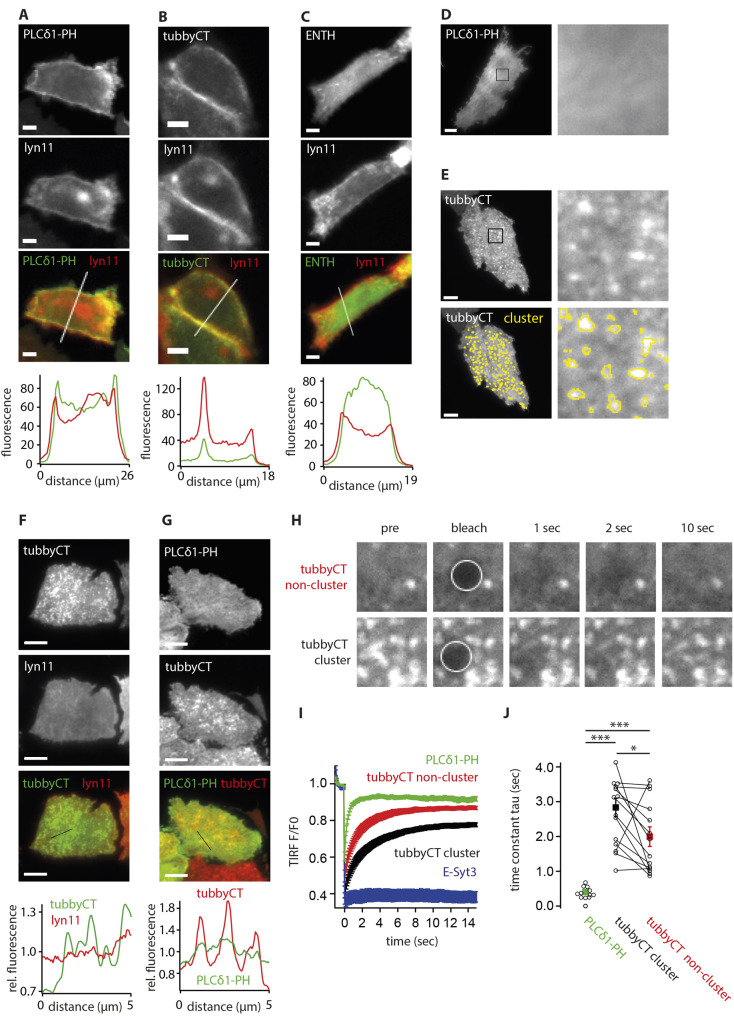
**Microdomain PM organization of the C-terminal domain of the tubby protein.** (A–C) Representative confocal images of CHO cells expressing the general PM marker Lyn11–RFP and the GFP-tagged PI(4,5)P_2_ sensors PLCδ1-PH (A), tubbyCT (B) and ENTH (C). Bottom, fluorescence intensity line profiles across lines indicated in merged fluorescence images demonstrate PM localization of PLCδ1-PH and tubbyCT, as opposed to ENTH localization. (D) Representative TIRF image of a CHO cell expressing PLCδ1-PH-GFP demonstrating homogeneous distribution. (E) Representative TIRF image of a CHO cell shows pronounced spatial clustering of GFP–tubbyCT. Yellow lines in lower panels indicate cluster areas as detected by the sectioning algorithm described in Methods. Right panels in D, E show enlargement of the rectangular regions indicated. (F) Representative TIRF image of a CHO cell co-transfected with GFP–tubbyCT and Lyn11–RFP indicates lack of clustering of Lyn11–RFP and lack of colocalization with tubbyCT. Bottom, fluorescence intensity line profiles for representative regions indicated in merged fluorescence images. Fluorescence intensities were normalized to mean value. (G) Colocalization analysis as in F. Cells were co-transfected with GFP–tubbyCT and PLCδ1-PH–GFP. Line profile plot reveals a slightly higher abundance of PLCδ1-PH in the tubbyCT cluster regions. Images in A–G from three experimental repeats. (H) Representative TIRF images of CHO cells expressing GFP-tubbyCT pre and post bleaching. White circles highlight the bleached areas. Note the delayed and incomplete recovery of tubbyCT clusters compared to homogeneously labeled PM. Size of image length: 3 µm. (I) Normalized time courses (mean±s.e.m.; PLCδ1-PH, *n*=11 cells; tubbyCT, *n*=14 cells; E-Syt3, *n*=9 cells) of FRAP experiments obtained from cells as in H and additionally from CHO cells expressing PLCδ1-PH–GFP and GFP–E-Syt3, respectively. (J) Time constants τ reveal decreased mobility of tubbyCT compared to PLCδ1-PH (***P<0.001; Dunnett's test: *P*<10^−4^). TubbyCT mobility was further reduced in the clusters compared to non-clustered regions (*P<0.05; paired two-tailed Student's *t*-test: *P*=0.039). Scale bars: 5 µm.

The GFP-tagged PI(4,5)P_2_ sensors TubbyCT and PLCδ1-PH predominantly localize to the PM (Santagata et al., 2001; Stauffer et al., 1998; Várnai and Balla, 1998). Accordingly, confocal imaging confirmed PM localization by coexpression with the general PM marker, Lyn11–RFP (Inoue et al., 2005) in CHO cells (Fig. 1A,B). In contrast, the low-affinity PI(4,5)P_2_ sensor ENTH–GFP predominantly localized to the cytoplasm and only showed a minor PM localization (Fig. 1A–C).

Considering the idea of spatially defined pools of PI(4,5)P_2_, we imaged the distribution within the PM of CHO cells of each of the sensors by TIRF microscopy, which facilitates the discrimination of PM subdomains due to greatly improved resolution in the *Z*-dimension. Strikingly, the PI(4,5)P_2_ sensors showed markedly distinct spatial distributions. While PLCδ1-PH distributed essentially homogeneously across the PM (Fig. 1D), tubbyCT displayed pronounced accumulation in clusters (Fig. 1E). ENTH weakly but homogeneously decorated the PM, with occasional bright accumulations most likely representing intracellular rodlike structures, as shown previously by confocal microscopy (Leitner et al., 2019). These structures had no spatial relationship to the PM clusters formed by tubbyCT (Fig. S1A).

We were intrigued by the domain-like organization of tubbyCT that, to our knowledge, has not been described before. TubbyCT clusters were immobile at the time scale of the imaging experiments (up to 4 min). Using a sectioning algorithm to quantify cluster size and area (see Materials and Methods), we found the clusters to occupy 10.5±0.9% of the imaged cell surface area with an average density of 0.66±0.03 per μm^2^ (mean±s.e.m.; *n*=57 cells). The same spatial distribution of tubbyCT was observed with an inverted GFP fusion construct, where GFP was fused to the C-terminus of the tubby domain (Fig. S1B–D).

Locally enhanced membrane fluorescence might result from locally increased membrane area. However, the membrane marker Lyn11 did not show co-enrichment, excluding that membrane folding or invagination caused the apparent tubbyCT clustering (Fig. 1F). Likewise, co-expression experiments of PLCδ1-PH and tubbyCT showed only minimal co-enrichment of PLCδ1-PH with the tubbyCT clusters (Fig. 1G).

To further characterize confinement into these domains, we used fluorescence recovery after photobleaching (FRAP), and compared diffusional mobility of tubbyCT in these domains and in the bulk membrane (Fig. 1H–J). A FRAP time course of tubbyCT was substantially slower compared to that of PLCδ1-PH (Fig. 1I,J), which appears to be consistent with the cooperative binding of tubbyCT, but not PLCδ1-PH, to the membrane via two PI(4,5)P_2_-binding sites, as discovered recently (Thallmair et al., 2022). Moreover, a recovery time course after photobleaching was slower and the immobile fraction of tubbyCT was significantly larger in the tubbyCT clusters compared to what was seen in non-cluster regions of the PM (Fig. 1I,J), indicating tighter association of tubbyCT with these structures compared to membrane binding in non-cluster regions of the PM.

Together, the findings suggest that tubbyCT specifically binds to PM s­ubdomains that are not preferentially recognized by the other two PI(4,5)P_2_-binding domains examined here.

### TubbyCT cluster formation depends on PI(4,5)P_2_

Given that the membrane association of tubbyCT is known to be mediated by binding to PI(4,5)P_2_, (Santagata et al., 2001; Thallmair et al., 2022) we first addressed the role of PI(4,5)P_2_ in cluster formation.

**Fig. 2.**
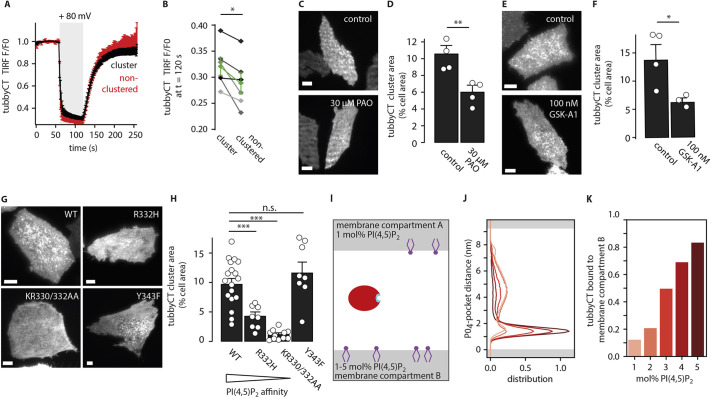
**PI(4,5)P_2_ is essential for recruitment of tubbyCT into PM clusters.** (A) GFP–tubbyCT membrane dissociation dynamics in clustered versus non-clustered regions induced by PI(4,5)P_2_ depletion by activation of co-expressed Ci-VSP. Fluorescence amplitude obtained from TIRF imaging of whole-cell voltage-clamped cells (mean±s.e.m.; *n*=6 CHO cells). (B) Comparison of residual tubbyCT membrane association after maximal Ci-VSP activation in the clustered and non-clustered regions, shown for the same individual cells analyzed in A with mean±s.e.m. shown in green (*P<0.05; paired two-tailed *t*-test, *P*=0.0275). (C) Representative TIRF images of GFP–tubbyCT membrane distribution in CHO cells under control condition and after 30 min incubation with PAO (30 µM). (D) Average cell surface area occupied by tubbyCT clusters (mean±s.e.m., control, *n*=16 cells, four experiments; PAO, *n*=14 cells, four experiments; **P<0.01; unpaired two-tailed Student's *t*-test: *P*=0.00824). (E) Representative TIRF images of GFP–tubbyCT membrane distribution in control CHO cells and after 10 min incubation with GSK-A1 (100 nM). (F) Average cell surface area occupied by tubbyCT clusters (mean±s.e.m., control, *n*=16 cells, four experiments; GSK-A1, *n*=12 cells, three experiments; *P<0.05; unpaired two-tailed Student's *t*-test: *P*=0.04110). (G) Representative TIRF images of CHO cells expressing GFP–tubbyCT wild-type (WT) and mutants R332H, KR330/332AA and Y343F. (H) Cell area occupied by GFP–tubbyCT clusters analyzed from images as in G. Mean±s.e.m., WT, *n*=39 cells, 18 experiments; R332H, *n*=33 cells, eight experiments; KR330/332AA, *n*=31 cells, 11 experiments; Y343F, *n*=19 cells, eight experiments. ***P<0.001; n.s., not significant; Dunnett's test: R332H, *P*=0.000382; KR330/332AA, *P*=1.92×10^−6^; Y343F, *P*=0.438). (I) Molecular dynamics simulations of tubbyCT binding to PI(4,5)P_2_ at the coarse-grained Martini level. Schematic simulation setup using a POPC bilayer (gray) doped with different fractions of PI(4,5)P_2_ (purple). In membrane compartment A, PI(4,5)P_2_ concentration was fixed to 1 mol%, in membrane compartment B, PI(4,5)P_2_ concentrations were adjusted from 1–5 mol%. TubbyCT (red) was initially placed in the water phase between both leaflets. To analyze the binding affinity, the distance between the PI(4,5)P_2_-binding pocket (cyan) and the PO_4_ plane of membrane compartment B was analyzed. (J) Histogram of the PO_4_-pocket distance using different concentrations of PI(4,5)P_2_ in membrane compartment B (1–5 mol% displayed from light to dark red). (K) Population of tubbyCT bound to compartment B. In case of the setup with 1 mol% PI(4,5)P_2_, the population average of both leaflets [which both contained 1 mol% of PI(4,5)P_2_] was employed. Scale bars: 5 µm.

Acute depletion of PI(4,5)P_2_ by the voltage-sensitive PI(4,5)P_2_ phosphatase from *Ciona intestinalis* (Ci-VSP) (Halaszovich et al., 2009; Rjasanow et al., 2015) provided direct evidence for an essential role of PI(4,5)P_2_ in tubbyCT clustering. Thus, acute activation of Ci-VSP released tubbyCT from the clusters, similar to dissociation of the domain from the bulk membrane (Fig. 2A). The fractional decrease of tubbyCT residence was slightly, but significantly, less compared to that in the bulk membrane (Fig. 2B), suggesting either an additional interaction contributing to clustering or limited access of Ci-VSP to its substrate in these structures.

Reduction in PI(4,5)P_2_ content in the PM by a pharmacological approach had a similar effect to that seen with Ci-VSP. PI(4,5)P_2_ is generated at the PM by sequential phosphorylation of phosphatidylinositol (PI) to PI(4)P, mainly catalyzed by PI4KIIIα, and subsequently to PI(4,5)P_2_ by PI4P5 kinases (Pemberton et al., 2019). We inhibited this pathway by incubation with well-characterized PI4 kinase inhibitors. Incubation with either the non-selective PI4 kinase inhibitor phenylarsine oxide (PAO) or the highly selective PI4KIIIα inhibitor GSK-A1 (Bojjireddy et al., 2014) strongly reduced clustering of tubbyCT (Fig. 2C–F), corroborating a role of PI(4,5)P_2_ in recruitment of tubbyCT to these compartments. Notably, PI4KIIIα inhibition (by incubation with GSK-A1) reduced localization of tubbyCT to clusters without strongly depleting overall PI(4,5)P_2_ concentration in the PM, which is in accordance with previously reported findings (Bojjireddy et al., 2014). Thus, loss of tubbyCT fluorescence was strongest in the clusters but only moderate in the other PM regions, leading to a more homogeneous PM association of tubbyCT (Fig. 2E,F; Fig. S2A–C). Furthermore, tubbyCT could still be released robustly from the membrane by induced depletion of PI(4,5)P_2_ with a PI-5-phosphatase after incubation with GSK-A1 (Fig. S2D,E), indicating substantial remaining PI(4,5)P_2_ levels after GSK-A1 treatment. Accordingly, the PM association of PLCδ1-PH–GFP was only slightly reduced by GSK-A1 (Fig. S2G,H), consistent with its higher PI(4,5)P_2_ affinity compared to tubbyCT (Thallmair et al., 2022).

Next, we analyzed compartmentalization of tubbyCT mutants with reduced PI(4,5)P_2_ affinity. In the R332H mutant, a semi-conservative mutation affects one of the positive charges in the canonical PI(4,5)P_2_-binding pocket (Santagata et al., 2001), which results in reduced PI(4,5)P_2_ affinity, as evidenced by poor membrane association (Fig. S3; also see Leitner et al., 2019; Quinn et al., 2008). As shown in Fig. 2G, tubbyCT(R332H) experienced a strongly reduced localization to clusters. Further reduction of PI(4,5)P_2_ affinity by the double mutation K330A and R332A (KR330/332AA) (Fig. S3), almost entirely abolished clustering (Fig. 2G,H). In contrast, a control mutation located within the lipid-binding pocket that did not substantially affect PI(4,5)P_2_ affinity (Fig. S3; Santagata et al., 2001) did not impact the clustering behavior (Fig. 2G,H). Mutations within the recently discovered second PI(4,5)P_2_-binding site (residues 301–305) of tubbyCT reduced clustering of tubbyCT in a similar manner to that seen with the R332H and KR330/332AA mutations (Thallmair et al., 2022).

Together, these observations indicate that binding to PM PI(4,5)P_2_ is essential for clustering of tubbyCT. We recently discovered that PM binding of tubbyCT involves a second PI(4,5)P_2_-binding site in addition to the previously known ‘canonical’ binding pocket (Thallmair et al., 2022). Cooperative binding through two binding sites steepens the PI(4,5)P_2_ concentration dependency of membrane association. Therefore, we wondered whether the tubbyCT clusters demarcate PM regions with locally increased PI(4,5)P_2_ concentrations that might not be strong attractors of PI(4,5)P_2_-binding domains that lack cooperative binding, such as PLCδ1-PH. To examine this possibility and to better understand how two membrane regions with different PI(4,5)P_2_ concentrations might affect tubbyCT localization, we performed coarse-grained molecular dynamics simulations. Specifically, we simulated binding of tubbyCT to membrane domains containing different PI(4,5)P_2_ concentrations (Fig. 2I) that compete for tubbyCT binding. Fig. 2J presents the observed distribution of membrane proximity of the canonical PI(4,5)P_2_-binding pocket of tubbyCT to two membrane compartments that have different PI(4,5)P_2_ concentrations. Notably, a moderately higher concentration (2–5 mol%) resulted in the supralinear accumulation of tubbyCT at this membrane relative to the second compartment containing a lower (1 mol%), but physiologically plausible concentration of PI(4,5)P_2_ (Fig. 2K). Notably, PLCδ1-PH, which binds PI(4,5)P_2_ through a single binding site, discriminated less between membrane compartments that differed in PI(4,5)P_2_ content (Fig. S4). Thus, moderately higher PI(4,5)P_2_ concentrations in PM subdomains could specifically attract tubbyCT, suggesting a mechanism for clustered distribution of tubbyCT. This conclusion is also consistent with the occasional slight enrichment of PLCδ1-PH at tubbyCT clusters (Fig. 1G).

### TubbyCT accumulates at E-Syt3-rich ER-PM junctions

Local accumulation of PI(4,5)P_2_ in the PM might arise from local PI(4,5)P_2_ synthesis. PI(4,5)P_2_ is mainly generated from PI via sequential phosphorylation by PI4Ks and PI4P5Ks. PI originates from the ER and is transferred to the PM at ER–PM junctions (Chang et al., 2013; Chang and Liou, 2015; Kim et al., 2015). In addition to PI(4,5)P_2_ being produced through this pathway, Golgi-derived PI4P is implicated in PI(4,5)P_2_ synthesis, particularly during continued activation of PLC (Myeong et al., 2021).

Hence, we examined for a spatial relationship of tubbyCT clusters with components of both pathways, by co-expressing either β-1,4-galactosyltransferase (residues 1–81), as a trans-Golgi marker (Yamaguchi and Fukuda, 1995), or the tether proteins extended synaptotagmin 1–3 (E-Syt1–E-Syt3), which are core components of ER–PM junctions (Giordano et al., 2013).

**Fig. 3.**
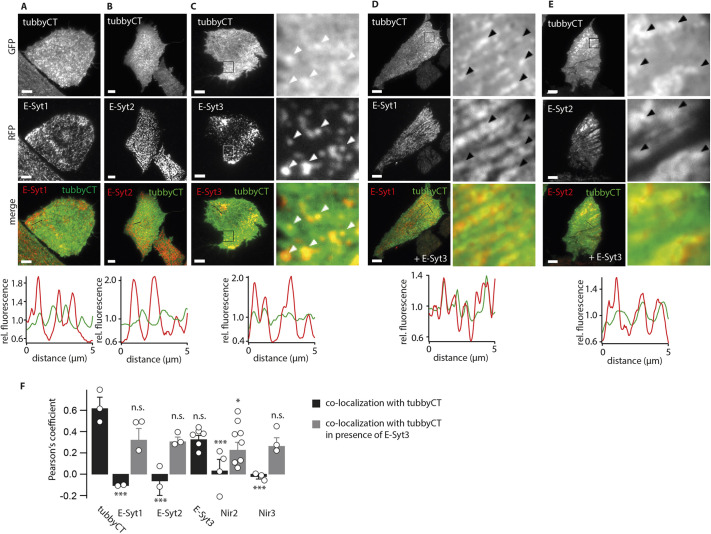
**TubbyCT localizes to ER-PM junctions.** (A–C) Colocalization analysis of GFP–tubbyCT with RFP–E-Syt1, RFP–E-Syt2 and RFP–E-Syt3, respectively. Transiently co-transfected CHO cells were imaged using TIRF microscopy. Lower panels, fluorescence intensity line profiles for representative regions indicated in merged fluorescence images above. Fluorescence intensities were normalized to mean value. Enlarged sections (right) in C are highlighted in overview images. (D,E) Colocalization analysis as in A–C. GFP–tubbyCT colocalized with RFP–E-Syt1 (D) and RFP–E-Syt2 (E), when CFP–E-Syt3 was additionally co-overexpressed. Arrowheads in C–E highlight areas of colocalizations. (F) Pearson's coefficient (mean±s.e.m.) from cells as in A–E and from colocalization analysis of GFP–tubbyCT with RFP–tubbyCT, Nir2–mCherry and Nir3–mCherry, respectively. Pearson's coefficient obtained from cells expressing GFP-tubbyCT with RFP/mcherry-tagged proteins only is shown in black, corresponding values from cells additionally expressing CFP-E-Syt3 are shown in gray. Mean±s.e.m. (tubbyCT, *n*=23 cells, three experiments; E-Syt1, *n*=16 cells, two experiments; E-Syt1+E-Syt3, *n*=25 cells, three experiments; E-Syt2, *n*=18 cells, two experiments; E-Syt2+E-Syt3, *n*=21 cells, three experiments; E-Syt3, *n*=26 cells, six experiments; Nir2, *n*=24 cells, three experiments; Nir2+E-Syt3, *n*=27 cells, eight experiments; Nir3, *n*=17 cells, three experiments; Nir3+E-Syt3, *n*=18 cells, three experiments). *P<0.05; ***P<0.001; n.s., not significant (Dunnett's test for multicomparisons to tubbyCT control was performed: E-Syt1: *P*=4.47×10^−5^, E-Syt1+E-Syt3: *P*=0.280, E-Syt2: *P*=2.35×10^−4^, E-Syt2+E-Syt3: *P*=0.0821, E-Syt3: *P*=0.0528, Nir2: *P*=8.50×10^−5^, Nir2+E-Syt3: *P*=0.0151, Nir3: *P*=4.96×10^−5^, Nir3+E-Syt3: *P*=0.0675). Scale bars: 5 µm.

TubbyCT did not colocalize with the trans-Golgi marker (Fig. S1E) or with the ER–PM junction markers E-Syt1 and E-Syt2 (Fig. 3A,B). However, tubbyCT colocalized with E-Syt3-enriched domains, as shown in Fig. 3C. Colocalization was quantified by calculating the Pearson's coefficient for pixel-wise correlation of fluorescence intensities (Fig. 3F). Thus, the coefficient was close to zero for correlation with E-Syt1 and E-Syt2, but ∼0.3 for E-Syt3. To validate this quantification, we attempted to calibrate the Pearsons's coefficient by examining experimental conditions where maximal colocalization is expected. Co-expression of GFP–tubbyCT with RFP–tubbyCT (Fig. 3F) or of two E-Syt isoforms known to form heteromers (Giordano et al., 2013), yielded even higher correlation coefficients of 0.6 to 0.8 (Fig. S5A–D). A more moderate correlation of tubbyCT with E-Syt3 was expected given that visual inspection already revealed a substantial fraction of membrane-localized tubbyCT outside of the tubbyCT clusters colocalized with E-Syt3. In contrast to tubbyCT, PLCδ1-PH did not substantially colocalize with any of the three E-Syt isoforms (Fig. S5E–H), although the correlation coefficients were slightly but consistently above zero for co-expression with E-Syt2 and E-Syt3, possibly reflecting the homogeneous distribution spanning the contact sites or the faint co-enrichment observed in co-expression with tubbyCT (Fig. 1G).

Given the colocalization with the ER–PM junction protein E-Syt3, we asked whether tubbyCT clusters in fact correspond to ER–PM junctions. Although lacking colocalization with co-expressed ER–PM tethers E-Syt1 and E-Syt2, upon additional overexpression of E-Syt3, tubbyCT also localized to the domains containing either E-Syt1–RFP (Fig. 3D,F) or E-Syt2–RFP (Fig. 3E,F), that is, to presumptive ER–PM junctions. We next examined colocalization of tubbyCT with additional junctional proteins. TubbyCT showed no obvious colocalization with the ER–PM components Nir2 and Nir3 (Chang and Liou, 2015; Kim et al., 2013, 2015) when co-expressed with RFP fusion constructs of these proteins. However, when E-Syt3 was additionally overexpressed, we observed colocalization to domains outlined by both Nir isoforms (Fig. 3F).

Taken together, we conclude that tubbyCT specifically assembles into ER–PM junctions that contain E-Syt3. This is a unique feature of tubbyCT not observed for the other PI(4,5)P_2_-binding domains examined here, namely PLCδ1-PH and ENTH.

### Clustering into ER–PM junctions is mediated by coincidence binding to E-Syt3 and PI(4,5)P_2_

**Fig. 4.**
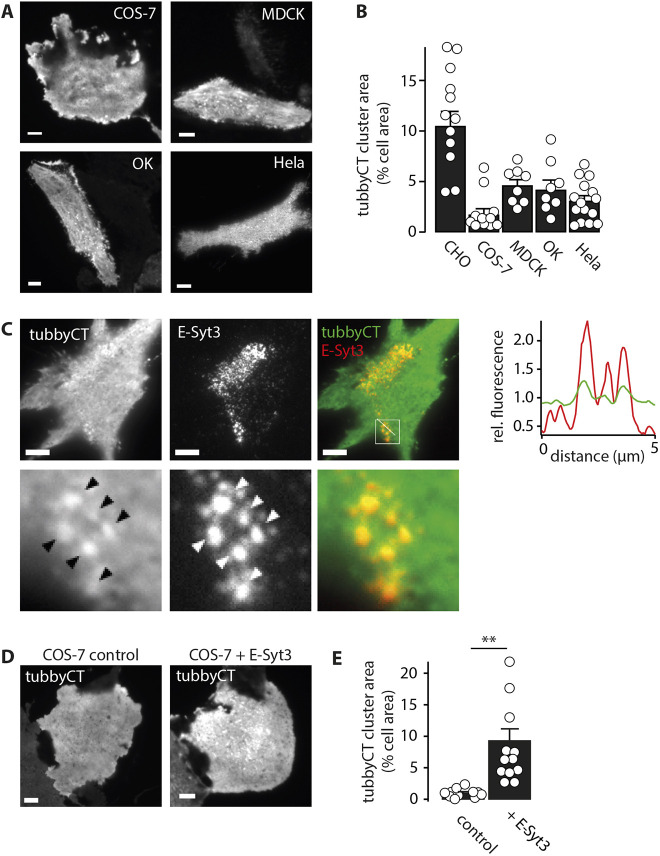
**The degree of TubbyCT clustering depends on cell type and can be induced by E-Syt3.** (A) Representative TIRF images of HeLa, MDCK, OK, and COS-7 cells transiently expressing GFP–tubbyCT. (B) Cell area occupied by GFP–tubbyCT clusters, quantified from TIRF images as in A. Mean±s.e.m. (CHO, *n*=43 cells, 12 experiments; COS-7, *n*=28 cells, 11 experiments; MDCK, *n*=31 cells, eight experiments; OK, *n*=37 cells, eight experiments; HeLa, *n*=46 cells, 15 experiments). (C) Colocalization analysis in HeLa cells reveals co-clustering of GFP–tubbyCT and RFP–E-Syt3. Right: line profiles as indicated in merged image. Fluorescence intensities are normalized to mean values. Arrowheads highlight areas of colocalizations. Images from three experimental repeats. (D) In COS-7 cells, E-Syt3 overexpression evoked tubbyCT cluster formation (right TIRF image; GFP–tubbyCT plus RFP–E-Syt3) not observed when tubbyCT was expressed together with free RFP (control; left). (E) Cell area occupied by tubbyCT clusters analyzed from images as in (D); mean±s.e.m. (control, *n*=21 cells, 11 experiments; +E-Syt3, *n*=21 cells, 12 experiments). **P<0.01 (unpaired two-tailed Student's *t*-test; *P*=0.00164). Scale bars: 5 µm.

To further address the role of E-Syt3 in compartmentalization of tubbyCT, we made use of the observation that the degree of clustering was different in different cell lines. Thus, in MDCK, OK and HeLa cells, clustering was detectable but less pronounced than in CHO cells, with tubbyCT clusters occupying ∼3–5% of the cell surface (Fig. 4A,B). Nevertheless, tubbyCT clusters colocalized with co-expressed E-Syt3, as shown for HeLa cells in Fig. 4C. In Cos-7 cells, tubbyCT clusters were barely detectable (Fig. 4A,B). However, when E-Syt3 was overexpressed, tubbyCT localization adopted the pronounced clustering characteristic for CHO cells, indicating that E-Syt3 is sufficient for recruiting tubbyCT into ER–PM junctions (Fig. 4D,E), independent of the cell type.

**Fig. 5.**
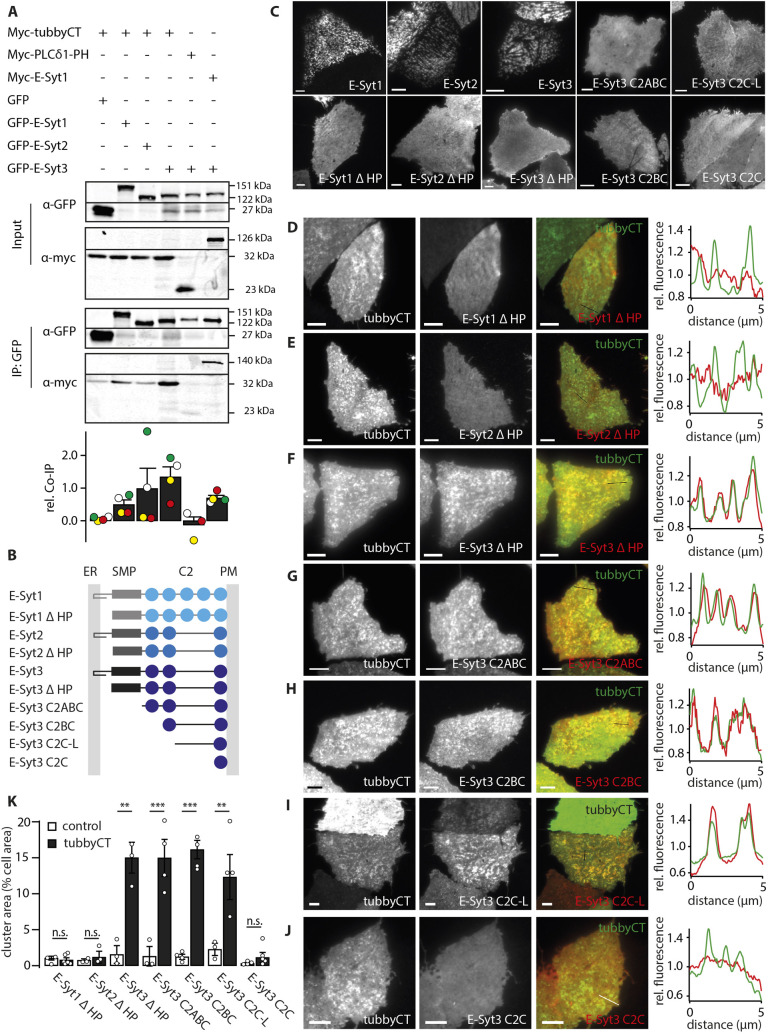
**Molecular interaction of tubbyCT and E-Syt3.** (A) Co-immunoprecipitation of Myc–tubbyCT with GFP-tagged E-Syt proteins. CHO cells were transfected with Myc- and GFP-tagged constructs as indicated. Protein lysates of cells co-expressing Myc-tubbyCT with free GFP and Myc–PLCδ1-PH with GFP–E-Syt3 were used as negative controls. Lysates of Myc–E-Syt1- and GFP–E-Syt3-expressing cells served as positive control. Representative blots of with anti-GFP and anti-Myc stained input and immunoprecipitation are shown. Lower panel shows quantification of four independent experiments (mean±s.e.m.). Input shows 15%. (B) Domain architecture of E-Syt isoforms and truncation constructs. (SMP domain, gray; C2 domains, blue; HP, ER membrane-binding hairpin). (C) Representative TIRF images of CHO cells transiently expressing the various RFP–E-Syt constructs shown in B. All truncation constructs exhibit uniform PM localization. (D–J) Representative TIRF images and colocalization analysis in cells co-expressing GFP–tubbyCT with the various RFP–E-Syt truncation constructs. Note rescue of clustered distribution and tubbyCT colocalization for E-Syt3 truncation constructs in F–I. (K) Cell area occupied by RFP–E-Syt clusters in presence of free GFP (control) and GFP–tubbyCT analyzed from images as in C–J. Mean±s.e.m. (E-Syt1ΔHP control, *n*=9 cells, four experiments; E-Syt1ΔHP+tubbyCT, *n*=16 cells, six experiments; E-Syt2ΔHP control, *n*=16 cells, three experiments; E-Syt2ΔHP+tubbyCT, *n*=15 cells, three experiments; E-Syt3ΔHP control, *n*=12 cells, three experiments; E-Syt3ΔHP+tubbyCT, *n*=15 cells, three experiments; E-Syt3C2ABC control, *n*=13 cells, three experiments; E-Syt3C2ABC+tubbyCT, *n*=17 cells, four experiments; E-Syt3C2BC control, n=15 cells, four experiments; E-Syt3C2BC+tubbyCT, *n*=17 cells, four experiments; E-Syt3C2C-L control, *n*=12 cells, three experiments; E-Syt3C2C-L+tubbyCT, *n*=15 cells, four experiments; E-Syt3C2C control, *n*=18 cells, four experiments; E-Syt3C2C+tubbyCT, *n*=18 cells, four experiments). **P<0.01, ***P<0.001; n.s., not significant (Scheffe test for multicomparisons). Scale bars: 5 µm.

Given that E-Syt3 promotes clustering of tubbyCT into ER–PM junctions, we next explored a potential direct interaction between both proteins. Co-immunoprecipitation (IP) experiments were performed with CHO cells transfected with Myc-tagged tubbyCT and GFP-fused E-Syt constructs. Fig. 5A shows that anti-GFP antibodies robustly co-precipitated Myc–tubbyCT from cells co-expressing GFP–E-Syt-3, and – to a lesser degree – from cells expressing GFP–E-Syt1 or GFP–E-Syt2. No interaction was observed between E-Syt3 and PLCδ1-PH. These findings are indicative for complex formation between tubbyCT and E-Syt3, and perhaps a weaker interaction with E-Syt1 and E-Syt2. However, co-precipitation of tubbyCT with the latter isoforms might as well result from indirect interaction mediated by endogenous E-Syt3, given the known dimerization of both E-Syt1 and E-Syt2 with E-Syt3 (Giordano et al., 2013). Thus, E-Syt1 and E-Syt2 likely co-precipitate tubbyCT bound to endogenous E-Syt3. We confirmed co-precipitation of E-Syt3 with E-Syt1 (Fig. 5A). Of note, this interpretation is also consistent with the finding that overexpression of E-Syt3 strongly enhances cellular colocalization of tubbyCT with E-Syt1 or E-Syt2 (Fig. 3).

In order to scrutinize such interaction and to localize the site of interaction within the modular domain architecture of E-Syt3, we examined colocalization of tubbyCT with various E-Syt truncation constructs. The E-Syts are anchored in the ER membrane by an N-terminal hairpin structure and bind to the PM with their C-terminal C2 domains (Giordano et al., 2013) as schematically shown in Fig. 5B. Accordingly, deletion of the hairpin sequence (E-Syt ΔHP) in any of the E-Syt isoforms resulted in a homogeneous distribution of the respective N-terminally RFP-labeled E-Syt in TIRF imaging (Fig. 5C). Thus, association with the ER and consequently with ER–PM junctions was lost (compare with Idevall-Hagren et al., 2015), but association with the PM persisted, likely mediated by the C2 domains.

Strikingly, co-expression of tubbyCT rescued clustering of E-Syt3ΔHP, but not E-Syt1ΔHP or E-Syt2ΔHP, into well-defined domains, where both proteins were closely colocalized (Fig. 5D–F,K). Thus, in a reverse manner, tubbyCT is capable of recruiting E-Syt3 into ER–PM junctions. This finding supports the direct interaction between tubbyCT and E-Syt3 in living cells, and further demonstrates that this interaction persists in the absence of the hairpin motif. In a series of further truncations of E-Syt3, tubbyCT-dependent clustering persisted when the SMP domain and additionally the first two (N-terminal) C2 domains (C2A and C2B) were deleted (Fig. 5G–K). Thus the minimum construct recruited into clusters by tubbyCT was a C-terminal region comprising the C2B-C2C-linker plus the C2C domain (Fig. 5I,K), whereas the C2C domain alone (including its C-terminal extension) was not sufficient for this interaction with tubbyCT (Fig. 5J,K). These experiments localize the interaction site for tubbyCT to the C2B- and C2C-linker region of E-Syt3.

As E-Syt3 PM binding is PI(4,5)P_2_ dependent, we assessed the effect of PI4K inhibition on E-Syt3 PM localization. Incubation of E-Syt3 expressing CHO cells in 100 nM GSK-A1 did not alter E-Syt3 distribution (Fig. S2I–K). Hence, the dependence of tubbyCT clustering on PI4K activity is directly driven by PI(4,5)P_2_ binding of tubbyCT and is not caused by a loss of E-Syt3 under these conditions.

Taken together, the dependence of tubbyCT clustering on PI(4,5)P_2_, its preferred residence in ER–PM junctions containing E-Syt3, and the facilitation of this localization by a direct interaction between tubbyCT and E-Syt3 indicate that a coincidence detection mechanism involving interaction with both E-Syt3 and PI(4,5)P_2_ mediates recruitment of tubbyCT to ER–PM junctions.

Given the binding to PI(4,5)P_2_ in the ER–PM junctions, we considered the possibility that tubbyCT overexpression could affect structure or function of these contact sites, either by shielding endogenous PI(4,5)P_2_ or contrariwise by enrichment through its cooperative PI(4,5)P_2_-binding properties. Imaging the distribution of E-Syts indicated that there was no change in either the area and number of the contact sites (Fig. S6A,D). Also, the PM association dynamics of E-Syt1 and E-Syt3 during PLC-mediated consumption of PI(4,5)P_2_ were unaltered when tubbyCT was overexpressed (Fig. S6B,C,E,F).

### Behavior of full-length tubby and TULP protein homologs

So far, we have studied the isolated tubby domain. We wondered whether association with ER–PM contact sites is also retained with the full-length tubby protein, and whether this property is a general feature of members of the tubby protein family.

**Fig. 6.**
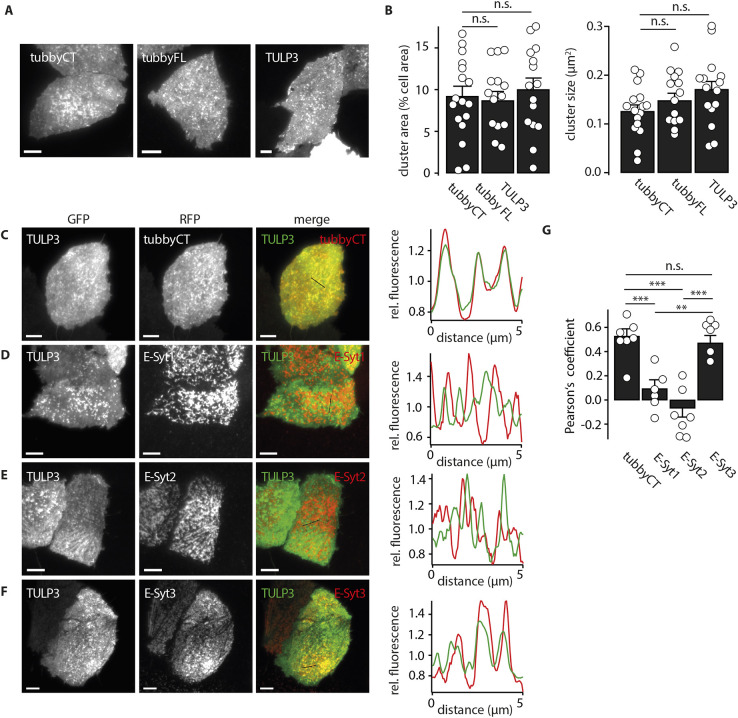
**Localization of TULP proteins to ER-PM junctions.** (A) Representative TIRF images of CHO cells expressing GFP–tubbyCT, full-length GFP-tubby (tubbyFL) or GFP–TULP3. (B) Cluster area (left panel) and cluster size (right) from CHO cells expressing the constructs shown in A. Mean±s.e.m. (tubbyCT, *n*=48 cells, 17 experiments; tubbyFL, *n*=37 cells, 14 experiments; TULP3, *n*=36 cells, 15 experiments). (Dunnett's test: cluster area, tubbyFL: *P*=0.991, TULP3: *P*=0.781; Dunnett's test: cluster size, tubbyFL: *P*=0.311, TULP3: *P*=0.078). (C) Colocalization analysis of GFP–TULP3 with RFP–tubbyCT. Representative TIRF images of transiently transfected CHO cell demonstrating that TULP3 occupies the same domains enriched in tubbyCT. Right panel, fluorescence intensity line profiles (normalized to mean intensities) for representative region indicated in merged fluorescence images. (D–F) Colocalization analysis as in C. CHO cells were co-transfected with GFP–TULP3 and RFP–E-Syt1 (D), RFP–E-Syt2 (E) or RFP–E-Syt3 (F), respectively. (I) Quantitative analysis of colocalizations shown in C–F using Pearson's coefficient (mean±s.e.m.; tubbyCT, *n*=23 cells, seven experiments; E-Syt1, *n*=16 cells, six experiments; E-Syt2, *n*=28 cells, seven experiments; E-Syt3, *n*=27 cells, six experiments). **P<0.01; ***P<0.001; n.s., not significant (Scheffe test for multicomparisons). Scale bars: 5 µm.

As shown in Fig. 6A,B, full-length tubby showed the same clustered distribution at the PM as the isolated tubbyCT domain, indicating that the native protein also preferentially localizes to ER–PM junctions.

Tubby is the founding member of the family of tubby-like proteins (TULPs). Notably, TULP1–TULP3 share the conserved C-terminal tubby domain (Mukhopadhyay and Jackson, 2011; Wang et al., 2018). We examined localization of TULP3, which has the most ubiquitous distribution across tissues in mammals (Mukhopadhyay and Jackson, 2011). TIRF imaging showed that in CHO cells, TULP3 has substantial PM localization which is organized in clusters highly similar to the distribution of tubby, both with respect to size and membrane area covered by the clusters (Fig. 6A,B). Furthermore, colocalization experiments with tubbyCT and with E-Syt isoforms (Fig. 6C–G) showed that TULP3 preferentially accumulates at E-Syt3-rich ER–PM junctions also populated by tubby.

In conclusion, PI(4,5)P_2_-dependent association with ER–PM junctions appears to be a general feature of TULPs, intriguingly suggesting they also serve a yet to be discovered function at these central cellular hubs of lipid and Ca^2+^ metabolism.

## DISCUSSION

### Readout of phosphoinositide signals by tubby proteins

The role of PI(4,5)P_2_ as a fundamental instructive signal for a plethora of cellular processes is well recognized (Balla, 2013; Di Paolo and De Camilli, 2006). Yet, binding to PI(4,5)P_2_ by recognition and effector proteins to influence manifold mechanisms must be specific with respect to space, time and biological context to produce meaningful cellular reactions. It has been pointed out before that this specificity is often achieved by coincidence detection, whereby PI(4,5)P_2_ effectors not only bind to PI(4,5)P_2_ via a recognition domain but simultaneously to additional cellular components to achieve proper localization and activity (Carlton and Cullen, 2005; Hammond and Balla, 2015).

The current findings add another twist to PI(4,5)P_2_ recognition by tubby and TULPs. Thus, in addition to cooperative lipid binding by two adjacent binding sites (Fig. 2; Thallmair et al., 2022), coincident binding to another protein, E-Syt3, introduces spatial specificity, recruiting tubbyCT preferentially into ER–PM junctions.

### TubbyCT as a sensor for junctional PI(4,5)P_2_

The current knowledge of the cell biology of phosphoinositides strongly relies on imaging approaches using genetically encoded biosensors to analyze the spatiotemporal profile of lipid concentration. Considering the broad relevance of PI(4,5)P_2_, as well the large number of proteins reported to bind to PI(4,5)P_2_, it is somewhat surprising that only very few PI(4,5)P_2_-binding domains have been used as PI(4,5)P_2_ biosensors. In fact, only few recognition domains with high specificity to PI(4,5)P_2_ have been identified (Hammond and Balla, 2015), and fewer have been characterized as a biosensor (Quinn et al., 2008; Szentpetery et al., 2009; Yoon et al., 2011). The above-mentioned coincidence detection strategies employed by PI(4,5)P_2_ effectors to mediate appropriate responses, might explain the apparent scarcity of high-specificity binding domains.

Most frequently, the PH domain of PLCδ1 has been used as a PI(4,5)P_2_ biosensor. Yet, the applicability of PLCδ1-PH can be compromised by its limited specificity, namely its high affinity to IP_3_. Thus, PLCδ1-PH might report on IP_3_ signals rather than PI(4,5)P_2_ concentration, especially during PLC signaling (for a comprehensive discussion, see Hammond and Balla, 2015). We recently characterized in some detail another structurally unrelated PI(4,5)P_2_ sensor domain, the ENTH domain from epsin, where membrane binding appears to depend on PI(4,5)P_2_ in a more straightforward manner (Leitner et al., 2019). However, ENTH–GFP suffers from low membrane residence at rest, limiting the signal-to-noise ratio, particularly in confocal microscopy.

Several studies have used tubbyCT as an alternative PI(4,5)P_2_ sensor (Hackelberg and Oliver, 2018; Hardie et al., 2015; Quinn et al., 2008), as its membrane association depends on PI(4,5)P_2_ concentration (Santagata et al., 2001), but is insensitive to IP_3_ (Leitner et al., 2019; Quinn et al., 2008). Here we show, that tubbyCT is a biased PI(4,5)P_2_ sensor; its localization is co-determined by binding both to PI(4,5)P_2_ in a cooperative manner and to the ER–PM protein, E-Syt3. These complex coincidence detection properties need to be taken into account when interpreting membrane association dynamics of tubbyCT in terms of PI(4,5)P_2_ concentration changes. It was noted previously that tubbyCT does not dissociate from the PM during PLC activity (e.g. Szentpetery et al., 2009), and this has been attributed to a high PI(4,5)P_2_ affinity. However, quantitative titration of PI(4,5)P_2_ by voltage-sensitive PI(4,5)P_2_ phosphatase (VSP) demonstrated unequivocally that its PI(4,5)P_2_ affinity is actually lower than that of the popular sensor domain PLCδ1-PH (Halaszovich et al., 2009; Leitner et al., 2019), which has been confirmed by molecular dynamics simulations (Thallmair et al., 2022). The recruitment of tubbyCT into ER–PM junctions mediated by interaction with E-Syt3 and PI(4,5)P_2_ might explain these seemingly contradictory findings.

Notwithstanding, we previously succeeded in using tubbyCT as a probe for neuronal PI(4,5)P_2_ dynamics in near-native hippocampal brain slice preparations. Different from its behavior in certain cell lines, tubbyCT readily dissociated from the PM following activation of muscarinic and glutamatergic Gq-coupled receptors, revealing neurotransmitter-dependent PI(4,5)P_2_ dynamics (Hackelberg and Oliver, 2018). Most likely, these neurons lack substantial expression of E-Syt3, which is consistent with RNA-seq data indicating predominant E-Syt2 expression in the hippocampal formation of the mouse, but little E-Syt3 (Sjöstedt et al., 2020).

Taken together, although genetically encoded sensors have been widely used and were instrumental in discovering many aspects of PI(4,5)P_2_ signaling and metabolism, quite surprisingly there is currently no well-characterized sensor that does not suffer from ambiguity in reporting PI(4,5)P_2_ concentrations.

On the other hand, preferential localization of tubbyCT might allow selective imaging of PI(4,5)P_2_ concentrations and dynamics in ER–PM junctions. To this end, we note the obvious potential to engineer versatile sensors for junctional phosphoinositides by fusing junctional targeting motifs to the various specific PI sensors that previously have been exploited for the examination of global PI dynamics. Indeed, engineered coincidence detectors for phosphoinositides revealed local PI dynamics at endocytic structures (He et al., 2017). There, a clathrin-binding module was fused to various PI-selective domains including PLCδ1-PH to successfully detect transient accumulation of PI(4,5)P_2_ during endocytosis events.

### An ER–PM junctional pool of PI(4,5)P_2_ ?

The question as to whether PM phosphoinositides are organized into distinct pools has attracted attention for a long time (reviewed in Doughman et al., 2003; Hammond, 2016). Experimental evidence includes both functional assays as well as fluorescence imaging approaches to address the spatial organization of PI(4,5)P_2_ (and other PIs). As an experimentally particularly well-supported example, PI(4,5)P_2_ synthesis from PI(4)P at the PM is largely independent of bulk PI(4)P, indicating a separate functional pool of PI(4)P designated for the synthesis of PI(4,5)P_2_ (Bojjireddy et al., 2014; Hammond et al., 2012, 2009; Nakatsu et al., 2012). Structurally, high-resolution light microscopy and electron microscopy using PI(4,5)P_2_-specific probes have demonstrated heterogeneity and even labeling of well-defined PI clusters (Fujita et al., 2009; van den Bogaart et al., 2011; Wang and Richards, 2012). However, these approaches generally used fixation protocols that potentially affected the lateral distribution of PIs, and other careful studies using live-cell approaches have failed to demonstrate substantial spatial heterogeneity (Hammond, 2016; van Rheenen et al., 2005).

With respect to ER–PM junctions, local enrichment of PI(4,5)P_2_ has been postulated before (Maléth et al., 2014), but direct evidence is lacking so far (Chang and Liou, 2016). ER–PM junctions are the primary site of delivery of the phosphoinositide precursor PI to the PM. Therefore, it is tempting to speculate that a local PI(4,5)P_2_ pool might be fed directly by PI delivered from the ER. However, it is not known whether ER–PM junctions are also a site of PI(4,5)P_2_ synthesis.

The clustering of tubbyCT discovered in this study provides some evidence in favor of the enrichment of PI(4,5)P_2_ in ER–PM junctions or a subset of these structures. First, higher tubbyCT occupancy of the junctions relative to bulk PM was dependent on PI(4,5)P_2_. Secondly, the pronounced reverse recruitment of truncated E-Syt3 lacking the ER anchor into the junctions by tubbyCT suggests an attraction sufficient for concentrating tubbyCT other than E-Syt3. Finally, differential recruitment of tubbyCT as opposed to PLCδ1-PH by PI(4,5)P_2_ can be attributed to distinct binding affinities and differential concentration dependence of binding (Thallmair et al., 2022). Although these findings are consistent with a contribution of local PI(4,5)P_2_ enrichment to attracting tubbyCT into ER–PM junctions, independent measures for PI(4,5)P_2_ concentrations at ER–PM junctions will be required. One caveat is that tubbyCT, through its cooperative PI(4,5)P_2_ binding, might induce or enhance the accumulation of PI(4,5)P_2_, thereby interfering with the spatial distribution of its ligand. Nevertheless, so far we found no direct indications for ‘artificial’ clustering of PI(4,5)P_2_ by overexpressed tubbyCT, inasmuch as the distribution of E-Syts and their dynamics upon receptor-induced consumption of PI(4,5)P_2_ were unchanged by co-expression of tubbyCT (Fig. S6). Specifically, Pacheco et al. (2023), recently showed unhindered diffusion of membrane-bound PI(4,5)P_2_-binding domains, including tubbyCT along the PM, including regions defined as ER–PM junctions as they contained E-Syt1. This appears at odds with the clustering and reduced mobility of tubbyCT observed in the present study. To this end, differences between cell types and heterogeneity of ER–PM junctions should be considered.

### Biological function of tubby localization to ER–PM junctions

The preferred localization to ER–PM junctions was not only observed with the isolated tubbyCT domain but was also prominent for the full-length tubby protein and for its homolog TULP3. This conserved behavior in the TULP family, as well as the specificity implied by interaction with E-Syt3 suggest a specific role related to the function of ER-PM junctions. While such a mechanism remains to be determined, we point out that TULPs might either be effectors of PI(4,5)P_2_ dynamics in the ER–PM contact site, or vice versa might have a role in controlling function, such as lipid metabolism, at these sites. However, addition of tubbyCT, did not immediately impact on ER–PM structure and function (Fig. S6).

So far, TULP proteins have been shown to have a well-established role in the selective transport of G-protein-coupled receptors (GPCRs), channels, enzymes and signaling proteins into cilia (Badgandi et al., 2017; DiTirro et al., 2019; Han et al., 2019; Legué and Liem, 2019; Sun et al., 2012). Mistargeting of GPCRs and the ensuing ciliary dysfunction can explain at least some of the phenotypes observed in the tubby mouse mutant, which include obesity, retinal degeneration and hearing loss (Mukhopadhyay and Jackson, 2011). Our new findings should stimulate the reconsideration of additional cellular mechanisms underlying the pathophysiology observed upon deletion of tubby family proteins.

Intriguingly, a recent study showed that E-Syt3 is specifically expressed in hypothalamic nuclei involved in energy homeostasis and food intake (Zhang et al., 2020), and thus shows a striking overlap with neuronal tubby expression (Kleyn et al., 1996). Moreover, E-Syt3 impacts on diet-induced obesity, where increased E-Syt3 levels promoted obesity whereas loss of E-Syt3 antagonized obesity (Zhang et al., 2020). Given the cellular interaction discovered in this study, both proteins might interact in the same pathway in regulating neuronal activity in energy homeostasis. In fact, both tubby and E-Syt3 have implicated in regulating proopiomelanocortin (POMC)-derived transmitters in this neural circuitry (Guan et al., 1998; Zhang et al., 2020). Although entirely speculative at the moment, it is tempting to propose that E-Syt3 might sequester tubby in these neurons and thereby downregulate its ciliary function, explaining why the effects of increased E-Syt3 abundance resemble the consequences of loss of tubby.

## MATERIALS AND METHODS

### Cell culture

CHO dhFr^−^ cells (CRL-9096, American Type Culture Collection) were cultured in MEM Alpha medium (Gibco, Thermo Fisher Scientific, Waltham, USA), COS-7 (kindly provided by A. Renigunta, Marburg), HeLa (kindly provided by R. Jacob, Marburg), OK (kindly provided by B. Fakler, Freiburg) and MDCK (kindly provided by R. Jacob) cells in DMEM GlutaMAX^TM^-I medium (Gibco), both supplemented with 10% fetal calf serum, 1% penicillin and 1% streptomycin. Cells were kept at 37°C and 5% CO_2_. Cells were tested negative for mycoplasm. They were seeded on cover slips (Fig. 1A–C), glass bottom dishes (Figs 2A–D,G,H and 4), or in glass bottom µ-slide VI^0.5^ flow chambers (ibidi, Martinsried, Germany; Figs 1D,E, 2E,F, 3, 5C–K and 6 and  Figs S1–S5) for imaging experiments and on polystyrene dishes for protein extraction. At 2 days after seeding, they were transfected using JetPEI^®^ DNA Transfection Reagent (CHO dhFr^−^ cells; Polyplus Transfection, Illkirch-Graffenstaden, France), JetPRIME^®^ DNA Transfection Reagent (HeLa cells; Polyplus Transfection) and Lipofectamine 2000 reagent (COS-7, OK and MDCK cells; Invitrogen, Carlsbad, USA). Experiments were performed 24 h (TIRF imaging) or 48 h (protein extraction) post transfection.

### Molecular biology

Expression constructs used for transfection were: human PLCδ1-PH [amino acids (a.a.) 1–170] in pEGFP-N1 (NM_006225.3); rat epsin1-ENTH (a.a. 1–158) in pEGFP-N1 (NM_057136.1); Ci-VSP in pRFP-C1 (AB183035.1); mouse tubbyCT (a.a. 243-505) in pEGFP-C, pEGFP-N1 and pRFP-C1 (NM_021885.4); mouse tubbyFL in pEGFP-C1 (NM_021885.4); Lyn11-FRB (a.a. 1–11 of Lyn; GCIKSKGKDSA) in pRFP-N1; human β-1,4-galactosyltransferase (a.a. 1–81) in pDsRed-Monomer (Clontech Laboratories, cat. no. 632480); human E-Syt1 in pEGFP-C1 (NM_015292), human E-Syt2 in pEGFP-C1 (NM_020728.2), human E-Syt3 in pEGFP-C1 (NM_031913.4), (Giordano et al., 2013); human Nir2–mCherry (Chang and Liou, 2015); human Nir3–mCherry (AB385472; Chang and Liou, 2015); human TULP3 in pGLAP1 (NM_003324.4; Badgandi et al., 2017), human M1R in pSGHV0 (NM_000738.2); pRFP-C1 and pEGFP-C1 as control vectors. RFP-tagged E-Syt1–E-Syt3 constructs were obtained by subcloning E-Syt1–E-Syt3 into pRFP-C3. CFP-tagged E-Syt3 construct was generated by fluorophore exchange of pEGFP-C1-E-Syt3 construct. The Myc tag was N-terminally added to tubbyCT via PCR amplification and sub-cloned into pcDNA3.1. Myc-PLCδ1-PH construct was generated by exchange of the tubbyCT portion of pcDNA3.1-myc-tubbyCT with PLCδ1-PH. N-terminally Myc-tagged E-Syt1 was achieved via tag exchange of pEGFP-E-Syt1. In all Myc-tagged constructs, the original start codon was removed by PCR. E-Syt1ΔHP (a.a. 125–1105), E-Syt2ΔHP (a.a. 106–846), E-Syt3ΔHP (a.a. 104–887), E-Syt3C2ABC (a.a. 296–887), E-Syt3C2BC (a.a. 439–887), E-Syt3C2C-L (aa 568–887) and E-Syt3C2C (a.a. 745–887) constructs were generated via PCR amplification from respective full-length constructs and subsequent sub-cloning into pRFP-C1 using BsrG1 and Sal1 (E-Syt1ΔHP), BglII and BamH1 (E-Syt2ΔHP) and HindIII and KpnI (E-Syt3 deletion constructs) restriction sites, respectively. pEGFP-C1-tubbyCT R332H and pEGFP-C1-tubbyCT Y343F were generated by using a QuikChange II XL Site-Directed mutagenesis kit (Stratagene, Agilent Technologies, Waldbronn, Germany). pEGFP-C1-tubbyCT KR330/332AA was generated by mutagenesis PCR using PfuUltra II Hotstart PCR Master Mix (Agilent Technologies, Santa Clara, USA). Rapamycin-induced PI(4,5)P_2_ depletion was performed with Lyn11-FRB (a.a. 1-11 of Lyn; GCIKSKGKDSA; a.a. 2026–2121 of FRB NM_004958.3) and CFP–FKBP–Inp54p (a.a. 30–108 of FKBP, NM_054014.3; Inp54p, Z74807) as described previously (Suh et al., 2006). All constructs newly generated in this study are available from the authors upon request.

### Co-immunoprecipitation

CHO dhFr^−^ cells expressing Myc- and GFP-fused constructs were washed in PBS and subsequently lysed in 50 mM Tris-HCl pH 7.2, 150 mM NaCl, 10 mM EDTA, 1% Triton X-100 and 1% protease inhibitor cocktail (Roche) for 30 min. Cell debris was separated from lysate by centrifugation (21,000 ***g***; 4°C; 20 min). 800 μg total protein was diluted in 600 µl dilution buffer (10 mM Tris-HCl pH 7.5, 150 mM NaCl and 0.5 mM EDTA) and used for immunoprecipitation (IP) by anti-GFP nanobodies covalently bound to agarose beads (GFP-Trap^®^_A, Chromotech, Planegg-Martinsried, Germany). 25 µl beads were washed three times in dilution buffer and then incubated with diluted cell lysates for 1 h at 4°C. Afterprotein binding, beads were isolated by centrifugation (2500 ***g***, 4°C, 2 min) and washed three times in dilution buffer followed by three washing steps in high Na^+^ buffer (10 mM Tris-HCl pH 7.5, 500 mM NaCl and 0.5 mM EDTA). After a final centrifugation (2500 ***g***, 4°C, 2 min), immunocomplexes were detached from beads by incubation in 2× Laemmli buffer [20% (v/v) glycerol, 100 mM Tris, 130 mM DTT, 6.5% (w/v) SDS, 0.013% (w/v) Bromphenol Blue] (95°C, 10 min). Supernatant was separated from beads by centrifugation (2500 ***g***, 2 min) and used for western blot analysis.

Myc- and GFP-tagged proteins were detected with rabbit polyclonal IgG anti-GFP (FL) (1:200; sc-8334 Santa Cruz Biotechnology, Dallas, USA) and mouse monoclonal IgG anti-c-Myc (1:200; sc-40 Santa Cruz Biotechnology) primary antibodies, and goat anti-rabbit-IgG (IRDye^®^ 800CW) (1:5000, 926-32211, Li-cor Biosciences, Bad Homburg, Germany) and donkey anti-mouse-IgG (IRDye^®^ 800CW) (1:5000, 926-32212, Li-cor Biosciences) secondary antibodies, respectively.

For each co-IP experiment, a negative control lysate of cells expressing Myc–tubbyCT only was added. For calculation of relative co-IP values, the band intensity of this negative control was subtracted from anti-Myc band intensities, and resulting co-IP values were normalized to immunoprecipitated anti-GFP band intensities.

### TIRF microscopy

Experiments shown in Figs 2A–D,G,H, and 4 were performed as previously described (Halaszovich et al., 2009). In brief, imaging was performed on a BX51WI upright microscope (Olympus, Hamburg, Germany) provided with a TIRF condenser (Olympus) and a LUMPlanFI/IR 40×/0.8 NA water immersion objective (Olympus). Laser excitation occurred at 488 nm (Picarro, Sunnyvale, CA, USA), emission light passed a 530 nm-long-pass emission filter and was detected by a CCD camera (TILL Photonics GmbH, Gräfelfing, Germany). Image acquisition was controlled by TILLvisION software (TILL Photonics GmbH).

All other TIRF experiments were performed on a Dmi8 upright microscope (Leica, Wetzlar, Germany) equipped with an Infinity TIRF module (Leica), a HC PL APO 100×/1.47 oil objective (Leica) and a widefield laser (Leica). GFP, RFP and CFP fluorescence was excited at 488 nm, 561 nm and 405 nm, respectively. Corresponding emission light passed GFP-T (505-555 nm), DS-Red-T (590-650 nm) and CFP-T (460–500 nm) emission filters (Leica). Images were acquired with an ORCA-Flash4.0 C13440-20C camera (Hamamatsu photonics, Hamamatsu, Japan) controlled by LAS X software (Leica).

During imaging cells were transfused with extracellular solution (5.8 mM KCl, 144 mM NaCl, 0.9 mM MgCl_2_, 1.3 mM CaCl_2_, 0.7 mM NaH_2_PO_4_, 5.6 mM D-glucose and 10 mM HEPES pH 7.4). In experiments combining TIRF microscopy and patch-clamping, images were taken every 4 s.

### FRAP

FRAP experiments were performed on the Dmi8 upright microscope (Leica, Wetzlar, Germany) equipped with an Infinity TIRF module (also see TIRF microscopy) and an Infinity Scanner (Leica). Images were taken every 100 ms, the bleached area was 1.0±0.2 µm^2^ in size. Fluorescence recovery traces were monoexponentially fitted using IGOR Pro software (WaveMetrics, Lake Oswego, OR, USA).

### Confocal microscopy

Experiments were performed on an upright LSM 710 Axio Examiner Z1 microscope equipped with a 100×/1.30 NA Oil UV objective (Carl Zeiss, Oberkochen, Germany). Images were acquired with Zen 2009 software (Carl Zeiss).

### Wide-field fluorescence microscopy

Experiments were performed on a Dmi8 upright microscope (Leica, Wetzlar, Germany). Images were acquired with an ORCA-Flash4.0 C13440-20C camera (Hamamatsu photonics, Hamamatsu, Japan) controlled by LAS X software (Leica). For determination of membrane localization of GFP–tubbyCT constructs, CHO cells were co-transfected with a membrane marker (Lyn11–RFP) or catalytically inactive Ci-VSP (RFP-Ci-VSP C363S). Line profiles across the cells were analyzed. GFP fluorescence intensities at membrane marker peaks were averaged and normalized to average cytosolic fluorescence.

### Patch clamp electrophysiology

Whole-cell patch clamp experiments for activation of Ci-VSP were performed as described previously (Halaszovich et al., 2009; Leitner et al., 2019). Briefly, voltage clamp recordings were done simultaneous to TIRF imaging with an EPC 10 amplifier controlled by PatchMaster software (HEKA Elektronik, Lambrecht, Germany). Patch pipettes were pulled from borosilicate glass (Sutter Instrument Company, Novato, CA, USA) and had an open pipette resistance of 2–3 MΩ after back-filling with intracellular solution containing (in mM) 135 KCl, 2.41 CaCl_2_, 3.5 MgCl_2_, 5 HEPES, 5 EGTA, 2.5 Na_2_ATP, 0.1 Na_3_GTP, pH 7.3 (with KOH), 290–295 mOsm/kg. Series resistance (R_s_) typically was below 6 MΩ. Cells were held at −80 mV and stepped to +80 mV to activate phosphatase activity of Ci-VSP for 60 s.

### Chemicals

The PI4 kinase inhibitors GSK-A1 (SYNkinase, Parkville, Australia) and phenylarsine oxide (PAO; Sigma-Aldrich, St Louis, USA) were dissolved in 1 mM (GSK-A1) and 100 mM (PAO) stock solutions in DMSO and diluted to their working conditions (100 nM GSK-A1; 30 mM PAO) in extracellular solution. Cells were incubated in PI4 kinase inhibitors for 10 min (GSK-A1) and 30 min (PAO) prior to imaging. Rapamycin was purchased as 5 mM solution in DMSO (Calbiochem, San Diego, USA) and diluted to a 1 µM working solution in extracellular solution (5.8 mM KCl, 144 mM NaCl, 0.9 mM MgCl_2_, 1.3 mM CaCl_2_, 0.7 mM NaH_2_PO_4_, 5.6 mM D-glucose, 10 mM HEPES pH 7.4).

### Data analysis

Image analysis was undertaken with ImageJ software. Pearson coefficients were calculated using Coloc2 plug-in.

A sectioning algorithm was used for tubbyCT cluster detection. For this, background subtracted images were median filtered (r=0.9 µm), subtracted from the background subtracted original image and locally thresholded using a Renyi entropy threshold. Pre-cluster regions of interest (ROIs) were generated from resulting binary images. For analysis of cluster size and cluster coverage of the cell, 10 images of a time series were taken into account. In Fig. 2A,B non-clustered bulk membrane was set with a distance of 1.8 µm to detected clusters.

Analysis and statistics of obtained imaging data was performed with IGOR Pro (WaveMetrics, Lake Oswego, OR, USA). Cells from one dish were assigned to one experiment. Mean values were calculated from individual cells, and the s.e.m. and statistics derive from the individual experiments. Data are displayed as mean±s.e.m.; additionally means of individual experiments are plotted as white circles on bar graphs. For comparison of two groups, unpaired two-tailed Student's *t*-tests were performed. Values derived from the same cells were analyzed using paired two-tailed Student's *t*-tests. For multicomparisons to one control condition, Dunnett's tests, or for general multicomparisons, Scheffe tests were performed. Asterisks indicate significance levels at *P<0.05, **P<0.01, ***P<0.001. Applied tests and *P*-values are also given in the figure legends.

### Coarse-grained molecular dynamics simulations

All simulations were performed using the open-beta version of the coarse-grained force field Martini (Souza et al., 2021; Souza and Marrink, 2020) and the program package Gromacs 2018.1 (Abraham et al., 2015). The proteins were described using a Gō-like model in combination with Martini (Poma et al., 2017; Souza et al., 2019). For tubbyCT, we used the crystal structure (PDB code: 1i7e; Santagata et al., 2001) and modeled the missing loops with I-TASSER (Yang et al., 2015). To generate the CG model, the missing loops were ligated to the crystal structure. This step was necessary to maintain the side chain orientations around the PI(4,5)P_2_-binding pocket of tubbyCT identified in the crystal structure. In case of the PLCδ1 PH domain, we built the CG model based on the crystal structure (PBD code: 1mai) using the resolved residues 12–130. The potential depth of the Lennard–Jones potentials of the Gō-like model was set to ε=12 kJ/mol. More details about the protein models can be found in Thallmair et al. (2022).

The simulation setup is schematically shown in Fig. 2I. The tubbyCT and the PLCδ1 PH domains, respectively, were placed in the water phase above a lipid bilayer consisting of POPC and a minor fraction of PI(4,5)P_2_. To study their competitive binding behavior with increasing PI(4,5)P_2_ concentration, one leaflet contained 1 mol% of PI(4,5)P_2_ in all systems, whereas the other leaflet contained concentrations ranging from 1–5 mol% PI(4,5)P_2_. The system was neutralized, and solvated in a 0.15 M solution of NaCl. After the equilibration (500 ps), three replicas of each setup were simulated for 50 µs each. Thus, a total simulation time of 750 µs was acquired for each protein.

To analyze the binding behavior of tubbyCT and PLCδ1 PH, the distance between the PO_4_ plane of the bilayer and the PI(4,5)P_2_-binding pocket was analyzed. Integration of the population up to a PO_4_-pocket distance of 2.25 nm and 2.0 nm y­ielded the membrane-bound fraction of tubbyCT and PLCδ1 PH domain, respectively.

## Supplementary Material

10.1242/joces.260848_sup1 Supplementary information
