# Supplementary material for: Localization of the tubby domain, a PI(4,5)P2 biosensor, to E-Syt3-rich endoplasmic reticulum–plasma membrane junctions
Source: J Cell Sci. 2023 Aug 4;136(15):jcs260848. doi: 10.1242/jcs.260848 (PMC10445746; doi:10.1242/jcs.260848)
Supplement: Supplementary information [file joces-136-260848-s1.pdf]

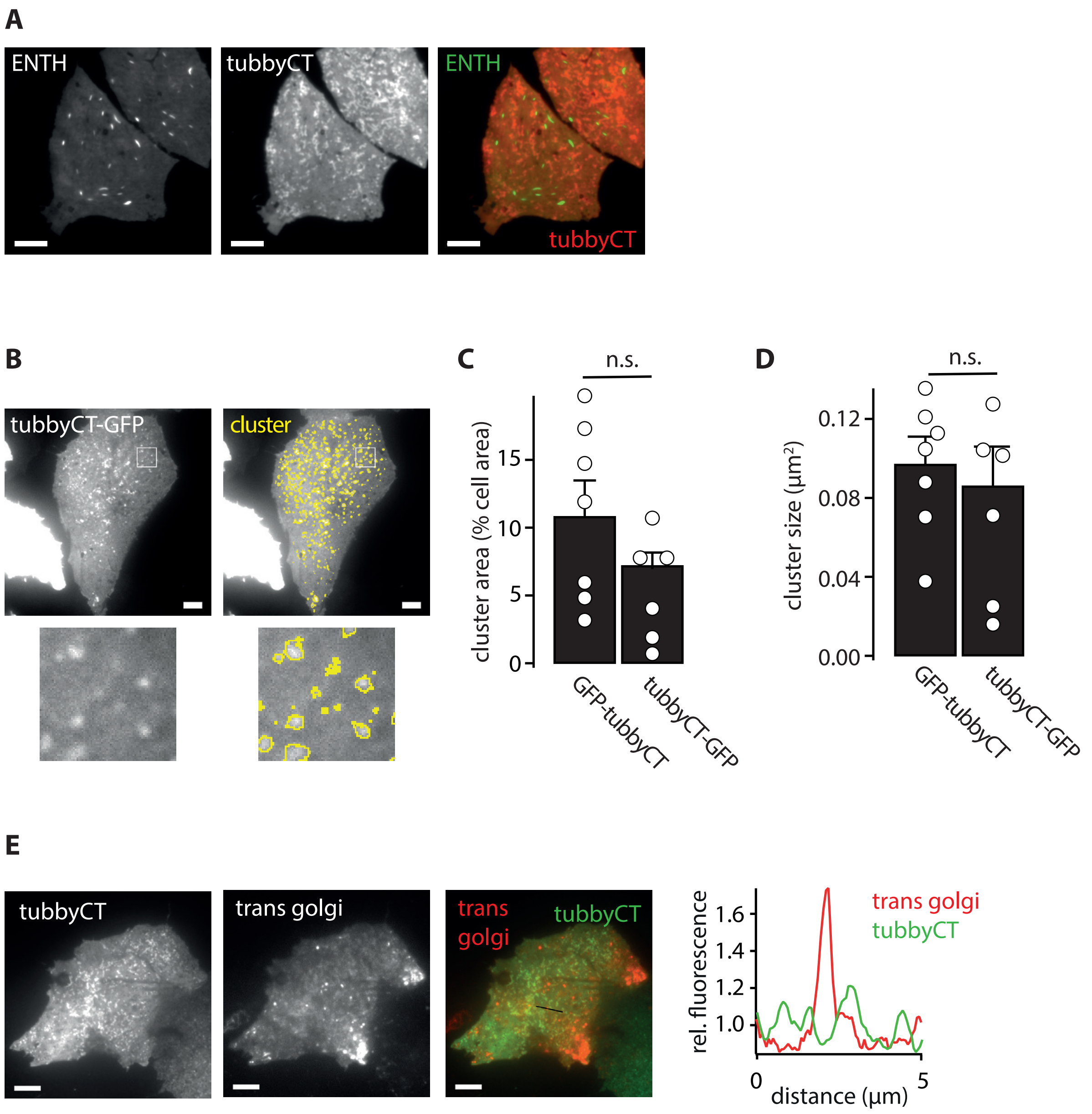

Fig. S1. No co-localization of tubbyCT with ENTH and trans golgi marker and clustering behavior of C-terminally GFP-fused tubbyCT

(A) Representative TIRF image of CHO cell co-transfected with RFP-tubbyCT and the PI(4,5)P2 sensor ENTH-GFP.

(B) Representative TIRF images of CHO cells expressing C-teminally GFP-tagged tubbyCT. Right, clusters are highlighted in yellow. Lower panels show enlargement of the rectangular regions indicated.

(C,D) Quantitative analysis of clustering of C- and N-terminally GFP-fused tubbyCT constructs from images as shown in (B). Student's test, cluster area: $p = 0.0852$; Student's test, cluster size: $p = 0.362$.

(E) Representative TIRF image of CHO cell co-transfected with GFP-tubbyCT and DsRed-tagged trans golgi marker beta 1,4-galactosyltransferase (AA 1-81). Fluorescence intensity line profiles for representative regions indicated in merged fluorescence image are shown on the right. Fluorescence intensities were normalized to the mean value.

Data is shown as mean ± SEM. Scale bars: 5 µm.

Journal of Cell Science • Supplementary information

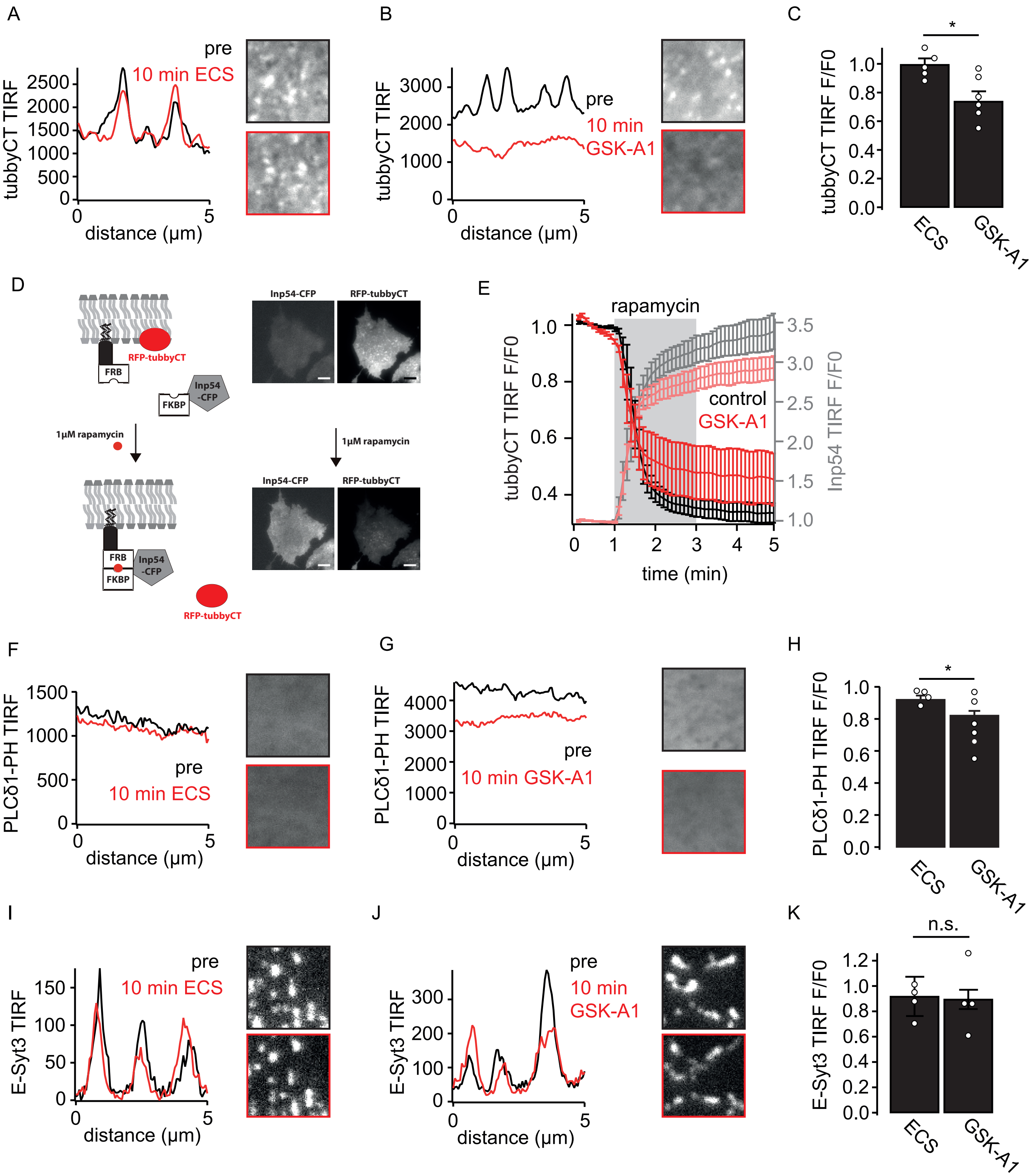

A
pre
10 min ECS
tubbyCT TIRF
distance (µm)
B
pre
10 min
GSK-A1
tubbyCT TIRF
distance (µm)
C
tubbyCT TIRF F/F0
ECS
GSK-A1
D
RFP-tubbyCT
FRB
Inp54
-CFP
FKBP
1µM rapamycin
Inp54-CFP
RFP-tubbyCT
E
rapamycin
control
GSK-A1
tubbyCT TIRF F/F0
Inp54 TIRF F/F0
time (min)
F
pre
10 min ECS
PLCδ1-PH TIRF
distance (µm)
G
pre
10 min GSK-A1
PLCδ1-PH TIRF
distance (µm)
H
PLCδ1-PH TIRF F/F0
ECS
GSK-A1
I
pre
10 min ECS
E-Syt3 TIRF
distance (µm)
J
pre
10 min
GSK-A1
E-Syt3 TIRF
distance (µm)
K
n.s.
E-Syt3 TIRF F/F0
ECS
GSK-A1

Fig. S2. Influence of PI4K inhibitor GSK-A1 on PI(4,5)P2 sensor domains and E-Syt3.
(A-C) CHO cells expressing GFP-tubbyCT were TIRF imaged pre and post 10 min incubation in extracellular solution (ECS, control) and 100nM GSK-A1, respectively. **(A)** Representative control images and line profiles of a CHO cell expressing GFP-tubbyCT. **(B)** Representative images and line profiles demonstrate loss of tubbyCT mainly from clustered but also from homogeneously labeled PM regions post incubation in GSK-A1
(C) Normalized tubbyCT fluorescence intensities reveal a significant decrease in PM localization upon incubation in GSK-A1 compared to control cells (student's t test, $p = 0.0154$). Image side lengths, 5 µm.
(D, E) PI(4,5)P2 dependence of tubbyCT PM binding post incubation in GSK-A1 was assessed with a recruitable 5'-phosphatase (Inp54-FKBP-CFP). **(D)** CHO cells transiently expressing a membrane anchored FRB domain (Lyn11-FRB), Inp54-FKBP-CFP and RFP-tubbyCT were incubated for 10 min in extracellular solution (control) and 100nM GSK-A1, respectively. Application of 1 µM rapamycin induces hetero-dimerization of FRB and FKBP and hence PM recruitment of Inp54. Example TIRF images of Inp54 PM recruitment and tubbyCT PM dissociation are shown. Scale bars, 5 µm. (E) TubbyCT (solid) and Inp54 (transparent) time courses of CHO cells incubated in extracellular solution (control) and GSK-A1, respectively.
(F-H) Experiments as in (A-C) performed with CHO cells expressing PLCδ1-PH-GFP reveal slight PM dissociation of PLCδ1-PH following GSK-A1 incubation. PM dissociation was significant compared to control cells (student's t test, $p = 0.0217$). Image side lengths, 5 µm.
(I-K) Experiments as in (A-C) performed with CHO cells expressing RFP-E-Syt3. PI4K inhibition does not alter E-Syt3 PM localization (student's t test, $p = 0.9467$). Image side lengths, 5 µm.

Journal of Cell Science • Supplementary information

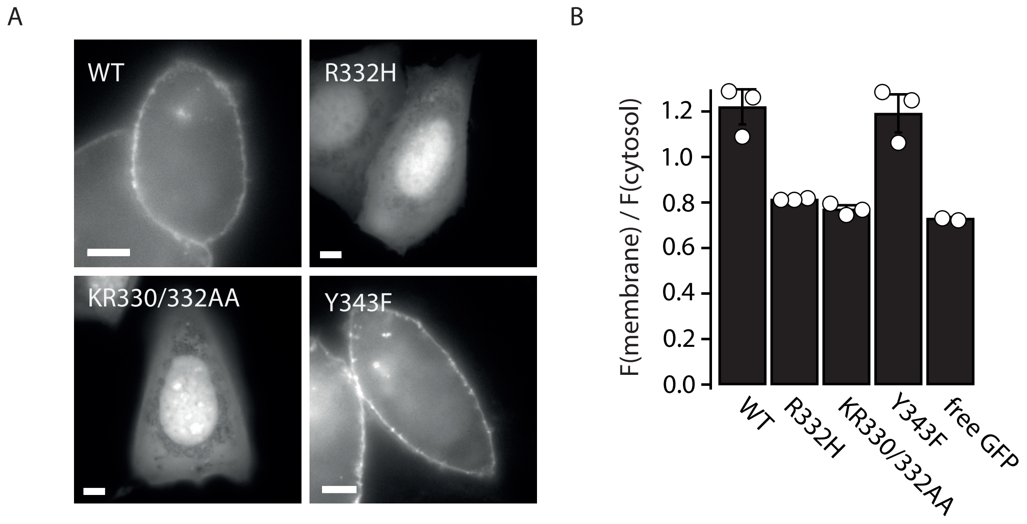

Fig. S3. PI(4,5)P2 affinity of tubbyCT mutants estimated from membrane association (A) Representative widefield fluorescence images of CHO cells expressing GFP-tubbyCT wild-type (WT) and mutants R332H, KR330/332AA, and Y343F. Note the different degrees of membrane association. Scale bars, 5 µm.

(B) Ratio of membrane-to-cytosolic fluorescence of the tubbyCT mutants and free GFP for comparison (mean ± SEM). Ratios were determined from line profiles with localization of the membrane determined as the local fluorescence maximum of RFP-CiVSP C363S co-expressed as a membrane marker (not shown).

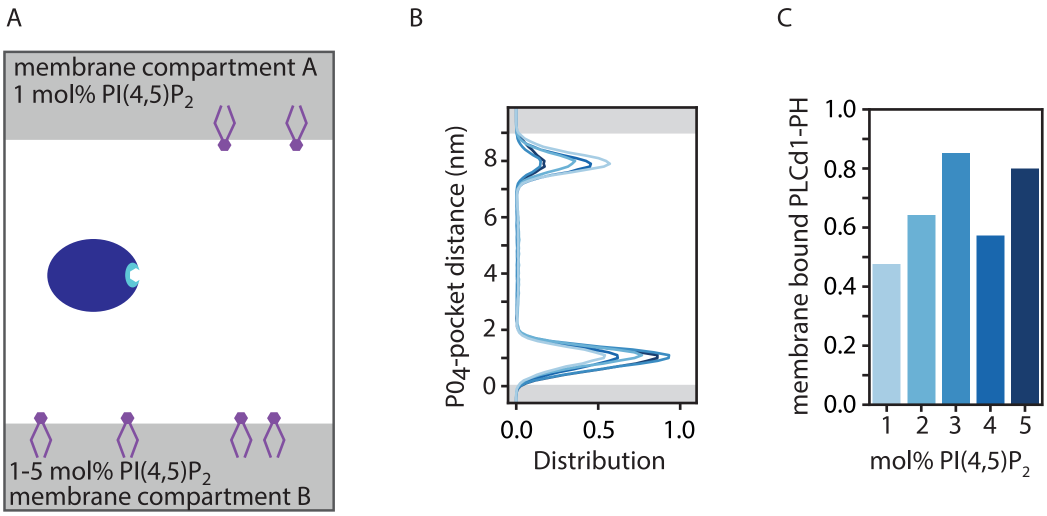

Fig. S4. Concentration dependent PI(4,5)P2 binding of PLCδ1-PH domain Molecular dynamics simulations of PLCδ1-PH binding to PI(4,5)P2 at the coarse-grained Martini level.**(A)** Schematic simulation setup using a POPC bilayer (grey) doped with different fractions of PI(4,5)P2 (purple). In membrane compartment A PI(4,5)P2 concentration was fixed to 1 mol%, in membrane compartment B PI(4,5)P2 concentrations were ranging from 1-5 mol%. PLCδ1-PH (blue) was initially placed in the water phase between both leaflets. To analyze the binding affinity, the distance between the PI(4,5)P2 binding pocket (cyan) and the PO4 plane of membrane compartment B was analyzed.
(B) Histogram of the PO4-pocket distance using different concentrations of PI(4,5)P2 in membrane compartment B (1-5 mol% displayed from light to dark blue).
(C) Population of PLCδ1-PH bound to compartment B. In case of the setup with 1 mol% PI(4,5)P2, the population average of both leaflets (which both contained 1 mol% of PI(4,5)P2) was employed.

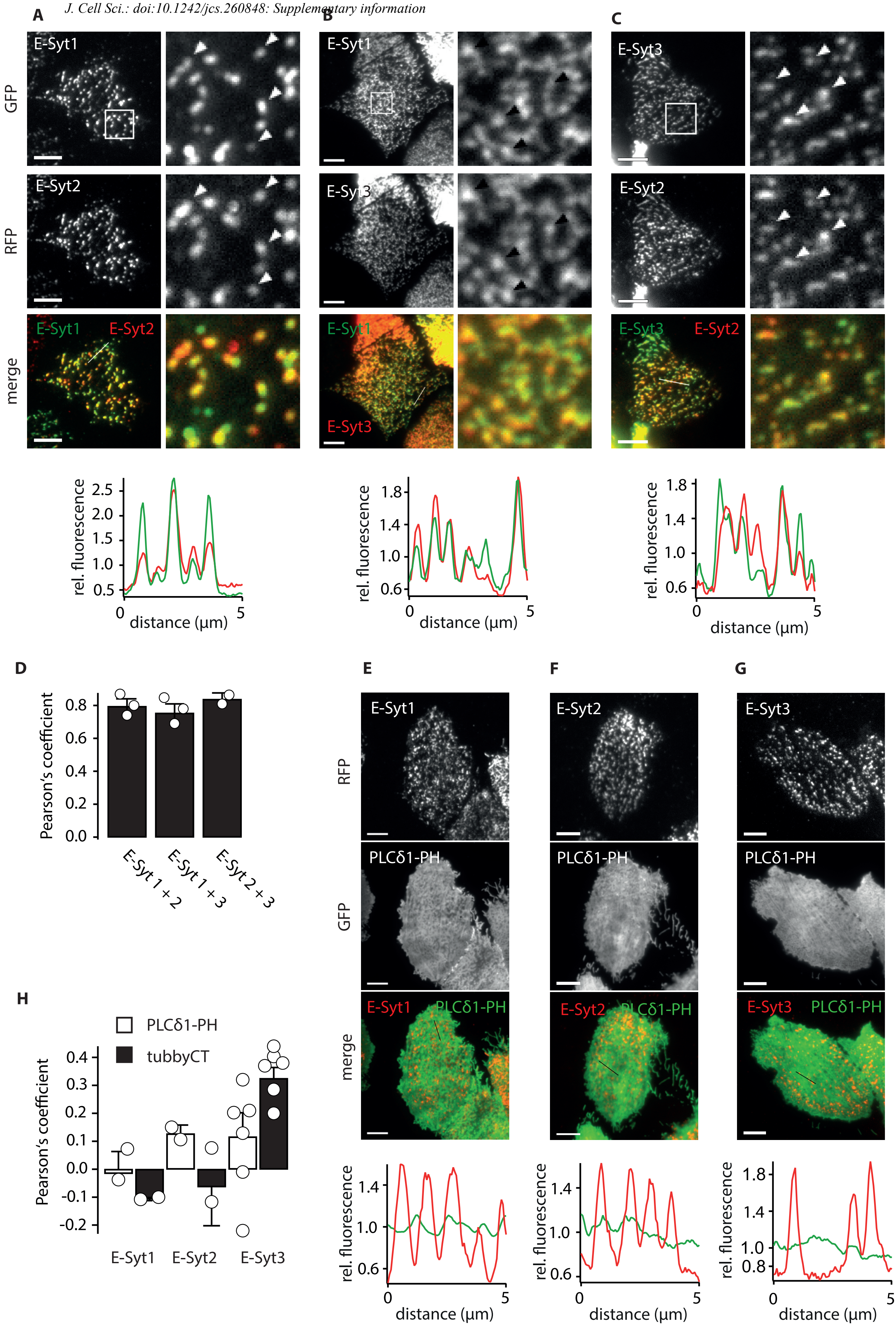

A
B
C
GFP
RFP
merge
E-Syt1
E-Syt2
E-Syt3
rel. fluorescence
distance (µm)
D
Pearson's coefficient
E-Syt 1+2
E-Syt 1+3
E-Syt 2+3
E
F
G
PLCδ1-PH
H
tubbyCT
E-Syt1
E-Syt2
E-Syt3

Fig. S5. Co-localization analysis of E-Syt isoforms among each other and with PLCδ1-PH

(A-C) Co-localization analysis of GFP-E-Syt1 co-expressed with RFP-E-Syt2 (A), GFP-E-Syt1 with RFP-E-Syt3 (B) and GFP-E-Syt3 with RFP-E-Syt2 (C). CHO cells were transiently transfected with the respective plasmids and imaged by TIRF microscopy. Enlarged sections (right) are highlighted in overview images. Bottom: Line profiles as indicated in merged images. Fluorescence intensities are normalized to mean values.

(D) Pearson's coefficients (mean ± SEM) from cells as in (A-C).

(E-G) Co-localization analysis of PLCδ1-PH-GFP with RFP-E-Syt1 (A), RFP-E-Syt2 (B) and RFP-E-Syt3 (C) was performed as described in (A-C).

(H) Pearson's coefficient (mean ± SEM) was obtained from images as shown in (E-G) (white). For comparison, data for GFP-tubbyCT colocalization are replotted from Figure 3F (black).

Scale bars, 5 µm.

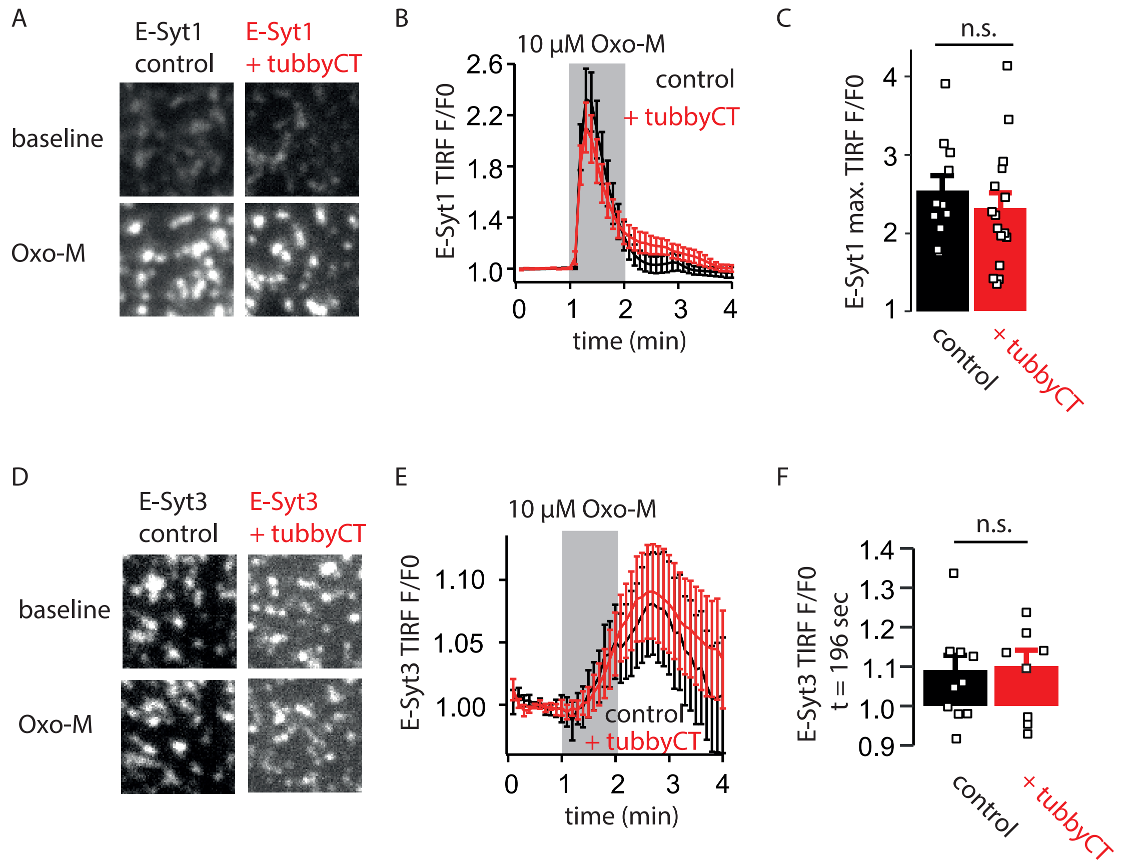

Fig. S6. TubbyCT does not infuence E-Syt1/3 localization and dynamics

(A) TIRF imaging of CHO cells co-expressing RFP-E-Syt1, M1R and free GFP (control) or GFP-tubbyCT, respectively. Representative images show RFP-E-Syt1 fluorescence before and maximal E-Syt1 fluore-scence during application of M1R agonist oxotremorine-M (Oxo-M, 10 µM).

(B) Normalized E-Syt1 TIRF fluorescences (mean ± SEM) from cells as in (A).

(C) Maximal normalized E-Syt1 fluorescences (mean ± SEM) obtained from time courses as shown in (B). E-Syt1 PM recruitmenr did not differ between control cells and cells co-expressing tubbyCT (student's t test, $p = 0.4117$).

(D-F) Experiments as in (A-C) from CHO cells co-expressing RFP-E-Syt3, M1R and free GFP (control) or GFP-tubbyCT, respectively. E-Syt3 dynamics were not changed in presence of tubbyCT compared to control cells (student's t test, $p = 0.5350$)

Image side lengths, 5 µm.
