# Supplementary material for: Localization of the tubby domain, a PI(4,5)P2 biosensor, to E-Syt3-rich endoplasmic reticulum–plasma membrane junctions
Source: J Cell Sci. 2023 Aug 4;136(15):jcs260848. doi: 10.1242/jcs.260848 (PMC10445746; doi:10.1242/jcs.260848)
Supplement: Supplementary file 1 [file joces-136-260848_review_history.pdf]

Localization of the tubby domain, a PI(4,5)P_2 biosensor, to E-Syt3-rich endoplasmic reticulum-plasma membrane junctions

Veronika Thallmair, Lea Schultz, Saskia Evers, Theresa Jolie, Christian Goecke, Michael G. Leitner, Sebastian Thallmair and Dominik Oliver
DOI: 10.1242/jcs.260848

Editor: Jennifer Lippincott-Schwartz

Review timeline
Original submission: 30 November 2022
Editorial decision: 17 January 2023
First revision received: 23 May 2023
Accepted: 26 June 2023

Original submission

First decision letter

MS ID#: JOCES/2022/260848

MS TITLE: Localization of the tubby domain, a PI(4,5)P2 biosensor, to E-Syt3-rich ER-PM junctions

AUTHORS: Veronika Thallmair, Lea Schultz, Saskia Evers, Theresa Jolie, Christian Goecke, Michael G. Leitner, Sebastian Thallmair, and Dominik Oliver
ARTICLE TYPE: Research Article

We have now reached a decision on the above manuscript.

To see the reviewers' reports and a copy of this decision letter, please go to: https://submit-jcs.biologists.org and click on the 'Manuscripts with Decisions' queue in the Author Area. (Corresponding author only has access to reviews.)

As you will see, the reviewers raise a number of substantial criticisms that prevent me from accepting the paper at this stage. They suggest, however, that a revised version might prove acceptable, if you can address their concerns. If you think that you can deal satisfactorily with the criticisms on revision, I would be pleased to see a revised manuscript. We would then return it to the reviewers.

Please ensure that you clearly highlight all changes made in the revised manuscript. Please avoid using 'Tracked changes' in Word files as these are lost in PDF conversion.

I should be grateful if you would also provide a point-by-point response detailing how you have dealt with the points raised by the reviewers in the 'Response to Reviewers' box. Please attend to all of the reviewers' comments. If you do not agree with any of their criticisms or suggestions please explain clearly why this is so.

© 2023. Published by The Company of Biologists under the terms of the Creative Commons Attribution License (https://creativecommons.org/licenses/by/4.0/).

<u>Reviewer 1</u>

Advance summary and potential significance to field

Thallmair et al. present data on the unique properties of the plasma membrane binding of the C-terminal domain of the Tubby protein (Tubby-CT). Tubby-CT has been described as PI(4,5)P2 binding domain that do not respond to Ins(1,4,5)P3 changes (in contrast to the widely used PH domain of PLCd1) and hence was recommended as a more faithful reporter of membrane levels of PI(4,5)P2. However, the Tubby domain displayed a behavior that suggested that it has perhaps too high affinity to PI(4,5)P2 and hence more resistant to follow PLC-induced changes in PI(4,5)P2. In a recent study, the Authors of the present work have described two PI(4,5)P2 binding sites in Tubby-CT that showed co-operativity. In the present study they show that the Tubby-CT signal is enriched in plasma membrane clusters that is more pronounced in some cells (such as CHO) and not in others (such as COS-7). They show that this enrichment is caused (at least partially) by the interaction of the domain with the ER-PM contact protein ESyt3 and also requires plasma membrane PI(4,5)P2 that is produced via the PI4KA-mediated PI4P formation. The Authors identified the region in the ESyt3 protein that is the site of interaction with the Tubby-CT. Lastly, they extend these observations to the other members of this family of proteins (Tubby like proteins or TULPs) and suggest that this unique feature may be important for their functions. The Authors also suggest that the Tubby-CT is a useful reporter of the PI(4,5)P2 pool that is associated with ER-PM contact sites.

These are potentially interesting studies that would attract significant interest from the lipid signaling community.

Comments for the author

There are several issues, however, that need attention before a final decision is made about suitability for publication in JCS.

Major points:

1. The major issue to be resolved is whether the PI(4,5)P2 enrichments in clusters described here are due to the enriched localization of Tubby-CT to ER-PM contact sites (by ESyt3) and keeping the lipid in those loci by the protein, or there are genuinely higher PI(4,5)P2 levels in these areas that contribute to the formation of Tubby-CT clusters. This reviewer is not convinced that the PI(4,5)P2 enrichment at these sites is the main driver of the Tubby-CT clusters. Rather, it is likely to be the opposite: Tubby-CT clusters by ESyt3 are the ones that maintain perhaps higher PI(4,5)P2 in those locations. While this sounds like a chicken-egg argument, it is an important one to address, as the Authors assertion is that Tubby-CT can monitor PI(4,5)P2 enrichments in the vicinity of contact sites. My reading of the data is that it is equally possible that the ESyt3 and Tubby-CT together generate the apparent PI(4,5)P2 enrichment at contact sites.

2. Along the same line: The only experiment that would suggest that Tubby-CT likes PI(4,5)P2-enriched areas at contact site is the ability of the domain to cluster an ESyt3 construct that misses its ER-anchor. This is a curious result and suggests that there are already endogenous ESyt3 in these cells that cluster Tubby-CT, which, in turn, can bring the truncated Esyt3 along. The finding that in some cell lines Tubby-CT does not show clustering, suggests that that those cells do not have sufficient ESyt3. Does this mean that those cells do not have PI(4,5)P2 enrichment in their ER-PM contacts? If the PI(4,5)P2 enrichment in the contacts depends on the presence of ESyt3 then a mutant Tubby-CT that cannot bind Esyt3 should still show clustering in cells expressing ESyt3.

3. One interesting and potentially important observation is the ability of PI4KA inhibitors to eliminate the Tubby-CT clusters. It would be important to know whether such treatment also affects the clustering of ESyt3 (if this was shown, I did not find it). Also, is there still even PM localization of the Tubby-CT after PI4KA inhibition that can be eliminated by PLC stimulation or 5-phosphatase activation? In other words, just the clusters are gone, and the general PM localization remains intact? These questions would be important to clarify in order to decide whether PI4KA inhibition changes the character of the ER-PM contacts or only eliminates the PI(4,5)P2 “enrichment” in those clusters.

© 2023. Published by The Company of Biologists under the terms of the Creative Commons Attribution License (https://creativecommons.org/licenses/by/4.0/).

4. A recent study (PMID: 36416724) showed that PI(4,5)P2 molecules (as bound to PLCd1-PH) freely diffuse in the PM even through ER-PM contacts. This paper was probably published after the submission of this work. However, it would be important to reconcile the suggestion put forward in this paper that PI(4,5)P2 is enriched (i.e. trapped to some extent) in ER-PM contact sites. Again, the parsimonious conclusion based on the presented data here is that the Tubby-CT is a coincidence detector that binds both PI(4,5)P2 and ESyt3 and this feature causes the clustering rather than a genuine PI(4,5)P2 enrichment that would be present even without ESyt3 and the expressed Tubby-CT.

All in all, I think this is an important and well-executed study but the conclusion that Tubby can monitor PI(4,5)P2 enriched domains at ER-PM contact sites should be critically discussed and perhaps toned down.

Reviewer 2

Advance summary and potential significance to field

In this manuscript, Thallmair et. al. addressed an question related to plasma membrane (PM) lateral inhomogeneity using a PI(4,5)P2 sensor, tubby CT. Specifically, they set out to understand how tubby CT clustered at the PM via imaging experiment and immunoprecipitation. They found that both tubby CT and full-length tubby protein accumulated at ER-PM junctions via interacting with E-Syt3. Overall, this is a well-designed study with good controls. Results are properly interpreted. They also did a good job preparing the manuscript in a coherent manner for the readers to follow their logic flow. The only concern I have is biological relevance of this work; it was not clear to me whether the authors wanted to steer this work as tool developing-validating or to emphasize the unique property of E-Syt3-rich ER-PM junctions. I believe this issue can be address editorially. There are a few addition suggestions as the following, but I believe this manuscript in its currently is suitable for publication in Journal of Cell Science.

Comments for the author

1. It would be nice to demonstrate the reduction of tubby CT clustering in E-Syt3 knockdown or knockout cells. This can be incorporated into Figure 3 or Figure 5.
2. For MD simulation in Figure 2, it would be more convincing to add a parallel analysis for PLCd-PH domain.

First revision

Author response to reviewers' comments

Response to reviewer #1

We thank the reviewer for the careful and constructive comments.

Most of the issues brought up center around the question, whether the tubbyCT clusters report on compartments that have an increased local PI(4,5)P_2 concentration and whether such local PI(4,5)P_2 inhomogeneities are native or rather induced by the overexpression and clustering of tubbyCT. Overall, we like to emphasize that the question of local PI(4,5)P_2 enrichment is not pivotal for our conclusions, which is accumulation of tubbyCT at ER-PM junctions by combinatorial binding to E-Syt3 and PI(4,5)P_2.

As we have only indirect evidence that tubbyCT reports on PI(4,5)P_2 enrichment at the contact sites, we toned down this point as suggested and explicitly discuss the caveat brought up by the

© 2023. Published by The Company of Biologists under the terms of the Creative Commons Attribution License (https://creativecommons.org/licenses/by/4.0/).

reviewer (see below). Moreover, we performed additional experiments including those suggested by the reviewer to further scrutinize the role of PI(4,5)P_2 in clustering of tubbyCT.

Below, we answer in a point-by-point manner to the detailed aspects of this theme.

1. *The major issue to be resolved is whether the PI(4,5)P2 enrichments in clusters described here are due to the enriched localization of Tubby-CT to ER-PM contact sites (by ESyt3) and keeping the lipid in those loci by the protein, or there are genuinely higher PI(4,5)P2 levels in these areas that contribute to the formation of Tubby-CT clusters. This reviewer is not convinced that the PI(4,5)P2 enrichment at these sites is the main driver of the Tubby-CT clusters. Rather, it is likely to be the opposite: Tubby-CT clusters by ESyt3 are the ones that maintain perhaps higher PI(4,5)P2 in those locations. While this sounds like a chicken-egg argument, it is an important one to address, as the Authors assertion is that Tubby-CT can monitor PI(4,5)P2 enrichments in the vicinity of contact sites. My reading of the data is that it is equally possible that the ESyt3 and Tubby-CT together generate the apparent PI(4,5)P2 enrichment at contact sites.*

As mentioned above, we think that the question of local enrichment of PI(4,5)P_2 is not a central point in our conclusions.

However, we agree that tubbyCT-induced enrichment should be considered and we now mention the possibility in the Discussion (p. 11). We also agree that, in the absence of additional independent measures of local PI(4,5)P_2, the suggestion of PI(4,5)P_2 -enriched regions at ER-PM junctions is somewhat speculative and we therefore now discuss this caveat explicitely (Discussion, p.13). Nevertheless, we still like to point out that preferential recruitment into domains with local PI(4,5)P_2 enrichment is consistent with and predicted by tubbyCT's unique cooperative binding of PI(4,5)P_2, discovered by us recently (Thallmair et al., 2022) and further explored in Fig. 2I-K, a feature not found in PLCd1-PH, (new additional Supplemental Figure 4). Thus PI(4,5)P_2 enrichment would be consistent with the differential behavior of tubbyCT versus PLCd1-PH.

One way of assessing the induction of PI(4,5)P_2 clustering by tubbyCT as proposed above would be to examine for structural or functional changes in ER-PM junctions as a consequence of tubbyCT over-expression, given that PM association of the ER-PM tethers, E-Syts, is mediated by PI(4,5)P_2. We thus performed additional experiments that analyze ER-junctional area (by imaging E-Syt1 and E-Syt3 distribution) as well as the E-Syt dynamics induced by activation of PLC by Gq-coupled GPCR. These data, now added to the manuscript as new Supplemental Figure S6, show no changes in ER-PM junction structure and behavior, thus failing at least to indicate a major impact of tubbyCT on junctional PI(4,5)P_2 concentrations.

2. *Along the same line: The only experiment that would suggest that Tubby-CT likes PI(4,5)P2-enriched areas at contact site is the ability of the domain to cluster an ESyt3 construct that misses its ER-anchor. This is a curious result and suggests that there are already endogenous ESyt3 in these cells that cluster Tubby-CT, which, in turn, can bring the truncated Esyt3 along. The finding that in some cell lines Tubby-CT does not show clustering, suggests that that those cells do not have sufficient ESyt3. Does this mean that those cells do not have PI(4,5)P2 enrichment in their ER-PM contacts?*

Our data clearly show that tubby association into ER-PM junctions requires PI(4,5)P_2 (Fig. 2), indicating that cooperative binding to PI(4,5)P_2 and E-Syt3 mediates the clustering into the ER-PM junctions. While such a mechanism does not necessarily require higher local PI(4,5)P_2 concentrations, the unexpected finding of recruitment of truncated E-Syt3 by tubbyCT, stimulated us to consider increased local PI(4,5)P_2. Initially, we also considered a mechanism suggested in the above reviewer's comment in which tubbyCT might recruit free truncated E-Syt3 by multivalent binding to native E-Syt3 in addition. However, we found such a mechanism too speculative, as this model requires either multiple E-Syt3 binding sites or tubbyCT-tubbyCT binding (oligomerization). There are no data favoring such interactions.

Following these consideration we had put forward the possibility that the ER-PM junctions populated by tubbyCT may feature local PI(4,5)P_2 enrichment. Differential E-Syt3 expression is an obvious candidate mechanism for the differences seen between cell lines. This could mean that also junctional PI(4,5)P_2 concentrations vary; however, since we do not know how PI(4,5)P_2 levels in

© 2023. Published by The Company of Biologists under the terms of the Creative Commons Attribution License (https://creativecommons.org/licenses/by/4.0/).

ER-PM contact sites are regulated, and whether E-Syt3 plays a role, we could only speculate. In any case, we think that it is reasonable to take heterogeneity of ER-PM junctions into account, both across and within particular cell types, as now mentioned in the manuscript.

If the PI(4,5)P2 enrichment in the contacts depends on the presence of ESyt3 then a mutant Tubby-CT that cannot bind Esyt3 should still show clustering in cells expressing ESyt3.

Eliminating E-Syt3 binding would indeed be a great way to sort out the relative contributions of E-Syt3 and PI(4,5)P_2 binding to tubbyCT clustering. However, we currently have no knowledge about the binding site, which prevents us from doing the suggested experiment.
However, we should emphasize that our data indicate that tubbyCT clustering is driven by both, E-Syt3 and PI(4,5)P_2.

3. One interesting and potentially important observation is the ability of PI4KA inhibitors to eliminate the Tubby-CT clusters. It would be important to know whether such treatment also affects the clustering of ESyt3 (if this was shown, I did not find it). In other words, just the clusters are gone, and the general PM localization remains intact? These questions would be important to clarify in order to decide whether PI4KA inhibition changes the character of the ER-PM contacts or only eliminates the PI(4,5)P2 "enrichment" in those clusters.

We did not look at that previously, but now performed additional experiments to answer these questions. The results are shown in the new Supplemental Figure S2I-K. We find no changes in E-Syt3 localization/clustering and membrane association following inhibition of PI4K, indicating that the reduced clustering of tubbyCT is indeed caused by lowered PI(4,5)P_2 levels at the junctions rather than by disruption of ER-PM junctions. At the same time, membrane association of the PH domain is only slightly, albeit significantly reduced by A1 (new Figure S2F-H). TubbyCT membrane association is reduced only partially by A1, notably most prominently at the clusters, as indicated by the loss of clustering reported initially (Figure2E,F; new Supplemental Figure 2A-C). Membrane binding of tubbyCT, however, is not abrogated by A1 treatment, as depletion of PI(4,5)P_2 by recruitment of PI(4,5)P_2 phosphatase (Rapamycin system) after A1 incubation still induces massive loss of membrane association of tubbyCT, that is only moderately attenuated compared to control conditions. (new Supplemental Figure 2D,E).
Together these new data support our conclusion that PI(4,5)P_2 binding strongly contributes to clustering of tubbyCT (at ER-PM junctions).

(As a side note: since A1 blocks resynthesis of PI(4,5)P_2 rather than directly eliminating PI(4,5)P_2, the differential effect of A1 on clustered tubbyCT versus bulk tubbyCT may indicate that at the clusters (i.e. ER-PM junctions) PI(4,5)P_2 turnover and/or synthesis is higher than in the bulk membrane. However, while we are currently further investigating this interesting idea, we think that this aspect is beyond the scope of the present manuscript).

4. A recent study (PMID: 36416724) showed that PI(4,5)P2 molecules (as bound to PLCd1-PH) freely diffuse in the PM even through ER-PM contacts. This paper was probably published after the submission of this work. However, it would be important to reconcile the suggestion put forward in this paper that PI(4,5)P2 is enriched (i.e. trapped to some extent) in ER-PM contact sites. Again, the parsimonious conclusion based on the presented data here is that the Tubby-CT is a coincidence detector that binds both PI(4,5)P2 and ESyt3 and this feature causes the clustering rather than a genuine PI(4,5)P2 enrichment that would be present even without ESyt3 and the expressed Tubby-CT.

We fully agree with this notion: the central conclusion of our paper is that coincident binding to E-Syt3 and PI(4,5)P_2 mediates clustering of tubbyCT at the junctions.
This said, such a mechanism should still result in reduced mobility of tubbyCT at ER-PM contact sites, which was not observed in Pacheca et al. To address this issue, we performed additional FRAP experiments quantifying tubbyCT mobility in clusters versus bulk membrane and indeed found reduced diffusional mobility in the clusters (new Figure 1H-J). We cannot explain the difference to the single-molecular diffusion measurements reported in Pacheco et al. with any certainty; however, given the cell type-dependent degree of clustering observed in our current study, we think that the different cell types studied may be the most likely explanation. Additionally, Pacheca et al. examined observed diffusion at sites enriched in E-Syt1 which, under resting

© 2023. Published by The Company of Biologists under the terms of the Creative Commons Attribution License (https://creativecommons.org/licenses/by/4.0/).

conditions is believed to not cluster at ER-PM junction, and moreover it is unclear if these sites contain E-Syt3.

Interestingly, a previous study found that septins, which significantly constrained diffusion of PI(4,5)P_2-bound sensor domains in the Pacheco et al. study, can form lipid diffusion barriers at ER-PM junctions (Sharma et al, Nature, 2013: 10.1038/nature12229) which may in fact reconcile the different observations on tubbyCT motility. However, this idea remains speculative, as septin abundance and localization with respect to ER-PM contacts has not been examined in either study.

Response to reviewer #2

We like to thank reviewer #2 for the helpful and positive comments.

1. It would be nice to demonstrate the reduction of tubby CT clustering in E-Syt3 knockdown or knockout cells. This can be incorporated into Figure 3 or Figure 5.

We agree that a knock-out of E-Syt3 could add additional evidence for the role of E-Syt3 interaction in clustering of tubbyCT.

Unfortunately, we did not succeed in generating a knock-out cell-line in the time available for review. We attribute the difficulties to the cell line from chinese hamster, were routine methods and precise genomic information were not directly available. However, we think that the complementary evidence we provide, i.e. induction of clustering by E-Syt3, biochemical interaction (CoIP) and the cellular assay that shows reverse clustering of E-Syt3 (but not other E-Syts) by tubbyCT, makes a strong case for the role of the interaction.

2. For MD simulation in Figure 2, it would be more convincing to add a parallel analysis for PLCd-PH domain.

As suggested, we performed the parallel MD simulation analysis for PLCd1-PH, and as expected found a much weaker discrimination between domains that differ in PI(4,5)P_2 content. This new data is now included as new Supplemental Figure 4, related to Figure 2.

Second decision letter

MS ID#: JOCES/2022/260848

MS TITLE: Localization of the tubby domain, a PI(4,5)P2 biosensor, to E-Syt3-rich ER-PM junctions

AUTHORS: Veronika Thallmair, Lea Schultz, Saskia Evers, Theresa Jolie, Christian Goecke, Michael G Leitner, Sebastian Thallmair, and Dominik Oliver
ARTICLE TYPE: Research Article

I am happy to tell you that your manuscript has been accepted for publication in Journal of Cell Science, pending standard ethics checks.

Reviewer 1

Advance summary and potential significance to field

This is a revised version of a previously reviewed manuscript. The Authors have addressed my criticisms by adding new experiments and expanding the Discussion.
These changes have significantly improved the manuscript. The characterization of the membrane association of the Tubby protein and the roles of PI(4,5)P2 and ESyt3 in the process is not only

© 2023. Published by The Company of Biologists under the terms of the Creative Commons Attribution License (https://creativecommons.org/licenses/by/4.0/).

important for advancing the field of biology of TULP proteins but also for better understanding the lipid organization of the plasma membrane.

Comments for the author

None

Reviewer 2

Advance summary and potential significance to field

The authors have addressed all my concerns. This manuscript is suitable for publication in JCS.

Comments for the author

No further comments

© 2023. Published by The Company of Biologists under the terms of the Creative Commons Attribution License (https://creativecommons.org/licenses/by/4.0/).
